# Supplementary material for: Association of dietary vitamin E intake with peripheral arterial disease: A retrospective cross-sectional study
Source: PLoS One. 2025 Mar 24;20(3):e0320356. doi: 10.1371/journal.pone.0320356 (PMC11932459; doi:10.1371/journal.pone.0320356)
Supplement: Attachment — Submitted filename: PONE-D-24-23667_R1_reviewer (2).pdf [file pone.0320356.s003.pdf]

PLOS ONE

Association of dietary vitamin E intake with peripheral arterial disease: A retrospective cross-sectional study

--Manuscript Draft--

Manuscript Number:	PONE-D-24-23667R1
Article Type:	Research Article
Full Title:	Association of dietary vitamin E intake with peripheral arterial disease: A retrospective cross-sectional study
Short Title:	Dietary Vitamin E Intake and Peripheral Arterial Disease
Corresponding Author:	Qiang Liu<br>Taiyuan City Central Hospital<br>Taiyuan, CHINA
Keywords:	dietary vitamin E intake; peripheral arterial disease; ankle-brachial index; National Health<br>and Nutrition Examination Survey; cross-sectional study
Abstract:	Introduction: The relationship between the amount of dietary vitamin E consumed and the development of peripheral arterial disease (PAD) remains a topic of debate. This relationship is of the utmost importance in the realms of healthcare and public health and is currently a highly researched and prominent topic.<br>This study aimed to gain deeper insights into the relationship between dietary vitamin E intake and PAD.<br>Methods: In a retrospective cross-sectional analysis, data from 6,588 participants in the National Health and Nutrition Examination Survey of the United States were examined during the period 1999–2004. We collected data on age, sex, race, marital status, education, physical activity, income, smoking, hypertension, diabetes, cardiovascular disease, body mass index, total cholesterol and HbA1c. Logistic regression and smooth curve fitting were used to support the research objectives.<br>Results: After accounting for all relevant factors, a negative correlation between dietary vitamin E intake and the likelihood of PAD was observed (OR: 0.981, 95% CI: 0.957–1.004). The overall prevalence of PAD was 5.9%, with 49.6% in males and 50.4% in females. Individuals in the third quartile of dietary vitamin E intake had a lower occurrence rate of peripheral artery disease than those in the first quartile (OR: 0.68, 95% CI: 0.51, 0.91). Similar patterns of association were observed in the subgroup analysis (all P values for interaction were >0.05).<br>Conclusions:Our study suggests a negative association between dietary vitamin E intake and incidence of PAD. Therefore individuals with insufficient dietary vitamin E intake, especially those with a very low intake, should consider increasing their vitamin E intake to lower the risk of developing PAD. These findings should be considered when offering dietary guidance to prevent PAD .
Order of Authors:	Qiang Liu
	Xing Wu
	Jianjun Shi
	Yun Wang
	Fei Zhao
	Xiang Wang
Response to Reviewers:	Dear Editor and Reviewers:<br>On behalf of my co-authors, we greatly appreciate the careful review and comments from both you and the reviewers. We believe that by implementing the suggested changes, we now have a stronger manuscript entitled “Association of dietary vitamin E intake with peripheral arterial disease: A retrospective cross-sectional study” for submission to Journal of PLOS ONE. We look forward to your positive response to the revised work submitted here.<br>We present here point-to-point responses for each of the comments in the attached

Powered by Editorial Manager® and ProduXion Manager® from Aries Systems Corporation

document and have revised our manuscript accordingly. We do not change our statistics or results. And we hope the revised manuscript could be acceptable to you. Revised sections are identified with red text in the paper.
There are no conflicts of interest regarding this work. All authors have read the revised manuscript and approved its submission to the journal “PLOS ONE”. Please do not hesitate to contact us if you have any questions.
Thank you and best regards.
Yours Sincerely,
Jianjun Shi
mail: 13934282361@163.com
Department of Cardiovascular Surgery,Taiyuan Central Hospital; Department of Cardiovascular Surgery, the Ninth Clinical College Affiliated to Shanxi Medical University, Taiyuan,030000, China.

Responds to the reviewer’s comments:

Reviewer #1:
Dear Reviewer,
Thank you very much for your time involved in reviewing the manuscript and your comments have greatly helped to improve the quality of our manuscript.

Comments: This is a population study whose methodology was presented in a coherent and well-founded manner, thereby providing a robust foundation for the authors' findings.The following considerations pertain to adjustments:
1. The acronym "ABI" should be written in full for the first time it is mentioned in the text.
2. While the methods of the primary study are duly referenced, it is nevertheless necessary to provide a detailed account of the methodology employed for the assessment of vitamin E consumption in this manuscript. Please indicate the number of 24-hour dietary recalls utilized in the study. Please clarify whether vitamin E intake was corrected for energy. It would be beneficial to ascertain whether the participants were taking a vitamin E supplement.
3. As noted, Reference 14 examined the same population and investigated the correlation between vitamin E intake and DAP. In what ways does your study differ from that of Reference 14?
4. The majority of the references utilized in this study are old, with 80% of them published more than five years ago. It is recommended that more current references be cited, particularly those used in the discussion to support the relationship between vitamin E consumption and DAP.
5. In line 228, the text makes reference to "recent studies," yet no corresponding reference is provided. Please provide the references for these recent studies.
6. The observational, cross-sectional study does not define a causal relationship. It is therefore advisable to exercise caution when considering the phrase "These findings should be used when formulating clinical guidelines."
7. The extant literature indicates that the isolated consumption of vitamin E does not provide cardiovascular protection. This is evidenced by the fact that studies that used supplementation did not observe the same results as observational studies. It is therefore of interest that this topic is introduced for discussion.

Comment 1:
The acronym "ABI" should be written in full for the first time it is mentioned in the text.
Respond 1:
Thank you for the valuable suggestion. Firstly, as suggested by you, the abbreviation ABI has been written in full and explained before its first use; Secondly, PAD is defined in the last sentence of the paragraph. As follows:

Ankle-brachial index

Systolic blood pressure measurements were taken from the right arm (brachial artery) and both ankles (posterior tibial artery) after a brief period of rest. If the participant's right arm measurements were unavailable due to any conditions that could affect accuracy, such as open wounds or dialysis shunts, the left arm was used for the brachial pressure measurement. Individuals aged 40-59 had their systolic blood

pressure measured twice, while those aged 60 and older had it measured only once. The ankle-brachial index (ABI) was calculated by dividing the average systolic blood pressure (ASBP) of the ankle by the ASBP in the arm. For participants aged 60 and older, the single reading represented the mean values. ABI <0.9 was used to define PAD.

Comment 2:
While the methods of the primary study are duly referenced, it is nevertheless necessary to provide a detailed account of the methodology employed for the assessment of vitamin E consumption in this manuscript. Please indicate the number of 24-hour dietary recalls utilized in the study. Please clarify whether vitamin E intake was corrected for energy. It would be beneficial to ascertain whether the participants were taking a vitamin E supplement.
Respond 2:
Thank you for the detailed review. Firstly,based on your this comment, we have added a description of the methodology used to assess vitamin E intake. Furthermore, in this study, the intake of vitamin E was not energy-adjusted, and this limitation has been addressed in the discussion. In addition, data related to vitamin E intake in diets was gathered from the NHANES 1999 to 2004 first-day dietary interview examination files. This interview captured details of all foods and beverages consumed within a 24-hour duration, such as consumption time, eating occasion, food descriptions, portion sizes, food sources, and location of consumption,and did not contain vitamin E supplement . As follows:

Data related to vitamin E intake in diets was gathered from the NHANES 1999 to 2004 first-day dietary interview examination files. A 24-hour dietary recall interview was conducted via the NHANES computer-assisted dietary data interview system to acquire detailed dietary intake data for all participants. This interview captured details of all foods and beverages consumed within a 24-hour duration, such as consumption time, eating occasion, food descriptions, portion sizes, food sources, and location of consumption. Following the dietary recall, a set of health-related inquiries were made. The data collection approach utilized the Automated Multiple Pass Method, which is the dietary data collection tool of the US Department of Agriculture (USDA).

Comment 3:
As noted, Reference 14 examined the same population and investigated the correlation between vitamin E intake and DAP. In what ways does your study differ from that of Reference 14?
Respond 3:
Thank you for the detailed review. The differences between my study and Reference 14 are as follows:
1. My study provides a research flowchart, demonstrating the design rationale of the study;
2. My study includes curve fitting graphs, making the study results more intuitive;
3. The covariates in my study include age, sex, race, marital status, education, physical activity, income, smoking, hypertension, diabetes, cardiovascular disease, body mass index, total cholesterol, and HbA1c, enhancing the reliability of the study;
4. My study further strengthens the stability of the results through subgroup analysis and forest plots. These are all significant improvements that can enhance the scientific rigor and credibility of the study.

Comment 4:
The majority of the references utilized in this study are old, with 80% of them published more than five years ago. It is recommended that more current references be cited, particularly those used in the discussion to support the relationship between vitamin E consumption and DAP.
Respond 4:
Thank you for the valuable suggestion. Based on your advice, the references in the study have been updated and supplemented, with literature published within the last 5 years accounting for 45.2%. However, the literature on the relationship between vitamin E and PAD published in the past 5 years is limited.
2.Zhang Z, Chen Z. Higher Systemic Immune-Inflammation Index is Associated With Higher Likelihood of Peripheral Arterial Disease. Ann Vasc Surg. 2022;84:322–6.
5.Zhu K, Qian F, Lu Q, Li R, Qiu Z, Li L, et al. Modifiable Lifestyle Factors, Genetic

Risk, and Incident Peripheral Artery Disease Among Individuals With Type 2 Diabetes: A Prospective Study. Diabetes Care. 2024;47:435–43.
6.Pan D, Guo J, Su Z, Meng W, Wang J, Guo J, et al. Association of prognostic nutritional index with peripheral artery disease in US adults: a cross-sectional study. BMC Cardiovasc Disord. 2024;24:133.
15.Hicks CW, Wang D, Matsushita K, McEvoy JW, Christenson R, Selvin E. Glycated albumin and HbA1c as markers of lower extremity disease inUS adults with and without diabetes. Diabetes
Res Clin Pract. 2022;184:109212.
16.Qu C-J, Teng L-Q, Liu X-N, Zhang Y-B, Fang J, Shen C-Y. Dose-Response Relationship Between Physical Activity and the Incidence of Peripheral Artery Disease in General Population:
Insights From the National Health and Nutrition Examination Survey 1999-2004. Front Cardiovasc Med. 2021;8:730508.
18.Liu Y, Chang L, Wu M, Xu B, Kang L. Triglyceride Glucose Index Was Associated With the Risk of Peripheral Artery Disease. Angiology. 2022;73:655–9.
19.Loreaux F, Jéhannin P, Le Pabic E, Paillard F, Le Faucheur A, Mahe G. An unfavorable dietary pattern is associated with symptomatic peripheral artery disease. Nutr Metab Cardiovasc Dis. 2024;34:2173–81.
20.Liu Y, Wei R, Tan Z, Chen G, Xu T, Liu Z, et al. Association between dietary fiber intake and peripheral artery disease in hypertensive patients. J Health Popul Nutr. 2024;43:118.
24.Cheng Y, Fang Z, Zhang X, Wen Y, Lu J, He S, et al. Association between triglyceride glucose-body mass index and cardiovascular outcomes in patients undergoing percutaneous coronary intervention: a retrospective study. Cardiovasc Diabetol. 2023;22:75.
25.Kumar M, Deshmukh P, Kumar M, Bhatt A, Sinha AH, Chawla P. Vitamin E Supplementation and Cardiovascular Health: A Comprehensive Review. Cureus [Internet]. 2023 [cited 2024 Mar 10]; Available from: https://www.cureus.com/articles/184881-vitamin-e-supplementation-and-cardiovascular-health-a-comprehensive-review.

Comment 5:
In line 228, the text makes reference to "recent studies," yet no corresponding reference is provided. Please provide the references for these recent studies.
Respond 5:
Thank you for the valuable suggestion. This sentence is redundant, it has been deleted.

Comment 6:
The observational, cross-sectional study does not define a causal relationship. It is therefore advisable to exercise caution when considering the phrase "These findings should be used when formulating clinical guidelines."
Respond 6:
Thank you for the valuable suggestion. This sentence is indeed not precise, it has been revised as follows: These findings should be considered when offering dietary guidance to prevent PAD.

Comment 7:
The extant literature indicates that the isolated consumption of vitamin E does not provide cardiovascular protection. This is evidenced by the fact that studies that used supplementation did not observe the same results as observational studies. It is therefore of interest that this topic is introduced for discussion.
Respond 7:
Thank you for the detailed review. I have already supplemented as per your request as follows:
Extensive clinical research has demonstrated the potential protective effects of vitamin E against long-term cardiovascular diseases[28]. Studies have indicated that taking vitamin E supplements for more than four years can result in a substantial 59% decrease in mortality from coronary disease[29]. The discussion regarding the impact of vitamin E on cardiovascular diseases is complex. While certain studies indicate that vitamin E supplements may provide protection against cardiovascular diseases, others do not support this claim. Additionally, a minority of studies have even suggested potential adverse effects[30,31]. So further investigations are necessary to validate our

	findings and explore the intricate relationships and potential mechanisms involved.<br><br>Reviewer #2:<br>Dear Reviewer,<br>Thank you very much for your time involved in reviewing the manuscript and your comments have greatly helped to improve the quality of our manuscript.<br><br>Comments: It is a well-written but incomplete article. Language editing is strongly recommended. Professional language support is recommended. In addition to the grammar check, the statistical as well as grammatical reasons why certain methods are favoured should be elaborated.<br>Respond：Thank you for the valuable suggestion. I have made the modifications to the grammatically incorrect parts as per your suggestion. Thank you once again for your guidance. After referring to a large number of previous literatures, the statistical methods used in this study include curve fitting, single factor analysis, multiple factor analysis, stratified analysis, subgroup analysis, etc.<br>1.Through the interpolation algorithm, curve fitting can generate a smooth curve between the given data points, thereby better understanding the trend and pattern of the data；<br>2.Single factor analysis not only helps in the initial exploration of the relationship between the predictor and response variables, aiding researchers in understanding the impact of individual variables on the outcome. It also assists researchers in selecting the variables that need to be included in the multiple factor analysis, for instance, by evaluating the changes in partial regression coefficients or OR values.<br>3.Univariate analysis primarily explores the impact of a single independent variable on the dependent variable through t-tests or analysis of variance (ANOVA). The t-test is suitable for comparing the means of two independent samples or paired samples, while ANOVA is used for comparing the means of three or more groups. These two methods are concise and straightforward, and can intuitively reveal the significant impact of a specific factor when acting independently on the outcome.<br>4.Multiple factor analysis uses multiple regression analysis to simultaneously examine the combined impact of multiple independent variables on the dependent variable and the interaction effects among the independent variables. This method can more comprehensively reflect the actual situation and reveal the hidden relationships behind complex data. Through multiple regression analysis, we can obtain the regression coefficients of each independent variable, thereby quantifying its specific contribution to the dependent variable, providing strong support for in-depth research.<br>5.Stratified analysis involves dividing the study subjects into strata based on one or more variables that need to be controlled, which can reduce the impact of confounding factors on the research results and thus provide a more accurate estimation of the association between the exposure factor and the outcome variable.<br>6.The main purpose of subgroup analysis is to study interactions or effect modification, i.e., whether the effect size varies in different populations or under different conditions. By dividing the study subjects into different subgroups based on certain characteristics (such as gender, disease severity, etc.), estimating the effect sizes for each subgroup separately, and conducting comparisons between subgroups, a better understanding of specific patient, intervention type, or study-specific issues can be achieved.
**Additional Information:**	
**Question**	**Response**
**Financial Disclosure**<br><br>Enter a financial disclosure statement that describes the sources of funding for the work included in this submission. Review the submission guidelines for detailed requirements. View published research articles from *PLOS ONE* for specific examples.	The author(s) received no specific funding for this work.

Powered by Editorial Manager® and ProduXion Manager® from Aries Systems Corporation

This statement is required for submission and **will appear in the published article** if the submission is accepted. Please make sure it is accurate.<br><br>**Funded studies**<br>Enter a statement with the following details:<br>• Initials of the authors who received each award<br>• Grant numbers awarded to each author<br>• The full name of each funder<br>• URL of each funder website<br>• Did the sponsors or funders play any role in the study design, data collection and analysis, decision to publish, or preparation of the manuscript?<br><br>Did you receive funding for this work?	
**Competing Interests**<br><br>Use the instructions below to enter a competing interest statement for this submission. On behalf of all authors, disclose any competing interests that could be perceived to bias this work—acknowledging all financial support and any other relevant financial or non-financial competing interests.<br><br>This statement is required for submission and **will appear in the published article** if the submission is accepted. Please make sure it is accurate and that any funding sources listed in your Funding Information later in the submission form are also declared in your Financial Disclosure statement.<br><br>View published research articles from *PLOS ONE* for specific examples.	The authors have declared that no competing interests exist.

**NO authors have competing interests**<br><br>Enter: *The authors have declared that no competing interests exist.*<br><br>**Authors with competing interests**<br><br>Enter competing interest details beginning with this statement:<br><br>*I have read the journal's policy and the authors of this manuscript have the following competing interests: [insert competing interests here]*<br><br>* typeset	
**Ethics Statement**<br><br>Enter an ethics statement for this submission. This statement is required if the study involved:<br><br>• Human participants<br>• Human specimens or tissue<br>• Vertebrate animals or cephalopods<br>• Vertebrate embryos or tissues<br>• Field research<br><br>Write "N/A" if the submission does not require an ethics statement.<br><br>General guidance is provided below. Consult the submission guidelines for detailed instructions. **Make sure that all information entered here is included in the Methods section of the manuscript.**	Study approval statement: The study did not require any additional institutional review board approval for the secondary analysis, thus ethical review and approval were exempted.<br>Consent to participate statement: The NHANES study was approved by the Ethics Review Committee of the National Center for Health Statistics (NCHS), and prior to their involvement, all participants provided written consent after being fully informed.

Powered by Editorial Manager® and ProduXion Manager® from Aries Systems Corporation

**Format for specific study types**<br><br>**Human Subject Research (involving human participants and/or tissue)**<br>• Give the name of the institutional review board or ethics committee that approved the study<br>• Include the approval number and/or a statement indicating approval of this research<br>• Indicate the form of consent obtained (written/oral) or the reason that consent was not obtained (e.g. the data were analyzed anonymously)<br><br>**Animal Research (involving vertebrate animals, embryos or tissues)**<br>• Provide the name of the Institutional Animal Care and Use Committee (IACUC) or other relevant ethics board that reviewed the study protocol, and indicate whether they approved this research or granted a formal waiver of ethical approval<br>• Include an approval number if one was obtained<br>• If the study involved *non-human primates*, add *additional details* about animal welfare and steps taken to ameliorate suffering<br>• If anesthesia, euthanasia, or any kind of animal sacrifice is part of the study, include briefly which substances and/or methods were applied<br><br>**Field Research**<br><br>Include the following details if this study involves the collection of plant, animal, or other materials from a natural setting:<br>• Field permit number<br>• Name of the institution or relevant body that granted permission	
**Data Availability**<br><br>Authors are required to make all data underlying the findings described fully available, without restriction, and from the time of publication. PLOS allows rare exceptions to address legal and ethical concerns. See the PLOS Data Policy and FAQ for detailed information.	Yes - all data are fully available without restriction

A Data Availability Statement describing where the data can be found is required at submission. Your answers to this question constitute the Data Availability Statement and **will be published in the article**, if accepted.<br><br>**Important:** Stating 'data available on request from the author' is not sufficient. If your data are only available upon request, select 'No' for the first question and explain your exceptional situation in the text box.<br><br>Do the authors confirm that all data underlying the findings described in their manuscript are fully available without restriction?	
**Describe where the data may be found in full sentences. If you are copying our sample text, replace any instances of XXX with the appropriate details.**<br><br>• If the data are **held or will be held in a public repository**, include URLs, accession numbers or DOIs. If this information will only be available after acceptance, indicate this by ticking the box below. For example: *All XXX files are available from the XXX database (accession number(s) XXX, XXX.).*<br>• If the data are all contained **within the manuscript and/or Supporting Information files**, enter the following: *All relevant data are within the manuscript and its Supporting Information files.*<br>• If neither of these applies but you are able to provide **details of access elsewhere**, with or without limitations, please do so. For example:<br><br>*Data cannot be shared publicly because of [XXX]. Data are available from the XXX Institutional Data Access / Ethics Committee (contact via XXX) for researchers who meet the criteria for access to confidential data.*<br><br>*The data underlying the results presented in the study are available from (include the name of the third party*	Publicly accessible datasets for this study can be found online. The name of the repository/repositories and their corresponding accession numbers are provided at http://www.cdc.gov/nchs/nhanes/ (accessed on 1 May 2022).

and contact information or URL).<br>• This text is appropriate if the data are owned by a third party and authors do not have permission to share the data.<br><br>* typeset	
Additional data availability information:	

Powered by Editorial Manager® and ProduXion Manager® from Aries Systems Corporation

Manuscript *手稿 Click here to access/download;Manuscript;Manuscript.docx

Research Article

Association of dietary vitamin E intake with peripheral arterial disease: A retrospective cross-sectional study

Qiang Liu[a,b,1], Xing Wu[c,d,1], Yun Wang[a,b], Xiang Wang[a,b], Fei Zhao[a,b], Jianjun Shi[a,b,*]

[a] Department of Cardiovascular Surgery, Taiyuan Central Hospital,China

[b] Department of Cardiovascular Surgery, the Ninth Clinical College Affiliated to Shanxi Medical University, China

[c] Department of Nephrology, Taiyuan Central Hospital, China

[d] Department of Nephrology, the Ninth Clinical College Affiliated to Shanxi Medical University, China

* Corresponding author

E-mail address: 13934282361@163.com

Keywords: dietary vitamin E intake; peripheral arterial disease; ankle-brachial index; National Health and Nutrition Examination Survey; cross-sectional study

Abstract

Background: The relationship between the amount of dietary vitamin E consumed and the development of peripheral arterial disease (PAD) remains a topic of debate. This relationship is of the utmost importance in the realms of healthcare and public health and is currently a highly researched and prominent topic.This study aimed to present the relationship between dietary vitamin E intake and PAD.

Methods: In a retrospective cross-sectional analysis, data from 6,588 participants in the National Health and Nutrition Examination Survey of the United States were examined during the period 1999–2004. We collected data on age, sex, race, marital status, education, physical activity, income, smoking, hypertension, diabetes, cardiovascular disease, body mass index, total cholesterol and HbA1c. Logistic regression and smooth curve fitting were used to support the research objectives.
Results: After accounting for all relevant factors, a negative correlation between dietary vitamin E

intake and the likelihood of PAD was observed (OR: 0.981, 95% CI: 0.957–1.004). The overall prevalence of PAD was 5.9%, with 49.6% in males and 50.4% in females. Individuals in the third quartile of dietary vitamin E intake had a lower occurrence rate of peripheral artery disease than those in the first quartile (OR: 0.68, 95% CI: 0.51, 0.91). Similar patterns of association were observed in the subgroup analysis (all P values for interaction were >0.05).

Conclusions: Our study suggests a negative association between dietary vitamin E intake and incidence of PAD. Therefore individuals with insufficient dietary vitamin E intake, especially those with a very low intake, should consider increasing their vitamin E intake to lower the risk of developing PAD. These findings should be considered when offering dietary guidance to prevent PAD .

Introduction

Peripheral arterial disease (PAD) is caused by atherosclerosis, a condition characterized by the accumulation of fatty deposits in the arteries of the lower extremities and feet[1]. It is prevalent disorder affecting over 200 million individuals worldwide [2]. In the United States, PAD affects around 8.5 million adults aged 40 years and above, with similar occurrence rates among men and women [3,4]. The economic and health burdens of PAD are substantial, emphasizing the need to identify its causes and implement appropriate measures. Previous studies have highlighted the significant roles of inflammatory markers, endothelial dysfunction, and oxidative stress in PAD development[5,6]. Although smoking, diabetes, hypertension, and dyslipidemia are established risk factors for PAD[3,7], the influence of diet is unclear. Further investigations are warranted to enhance our understanding of preventive measures and management strategies for PAD, given the limited knowledge of effective preventive factors.

Vitamin E, an antioxidant that dissolves in fat, is recognized as a key player in reducing lipid peroxidation in experimental settings[8]. Gey [4] first proposed the idea of vitamin E's potential in preventing cardiovascular disease in the "antioxidant hypothesis of atherosclerosis". Subsequent evidence linked vitamin E to atherosclerosis and thrombotic complications, attributing its anti-atherosclerotic activity to a range of biological functions, including antioxidant functions as scavengers of free radicals, in addition to non-antioxidant roles, such as controlling signal transduction, cellular proliferation, and gene expression. Vitamin E has attracted interest because of its anti-atherogenic and antioxidant properties, which have been associated with improved cardiovascular health in numerous studies. There was a significant increase in alpha-tocopherol levels [9–11].However while some studies indicate that supplemental vitamin E may offer protection against cardiovascular diseases, others do not, and a few even suggest the potential for adverse effects[12,13].

Therefore, to address this knowledge gap, the ultimate goal was to present a deeper understanding of how dietary vitamin E intake influences PAD and the implications of this relationship. We sought to gain deeper insights into the relationship between dietary vitamin E intake and PAD through a retrospective cross-sectional study involving 6,588 participants in the National Health and Nutrition Examination Survey (NHANES). We aimed to provide valuable information for personalized nutritional management strategies and contribute to a deeper understanding of the nutritional status of individuals and populations.

Methods

Study population

During a retrospective cross-sectional analysis, data from 6,588 participants in the NHANES of the United States were examined during the period 1999–2004[14–16]. The NHANES compiles diverse health-related data, including demographic details, physical examination outcomes, laboratory results, and dietary patterns. The National Center for Health Statistics collected this information with the approval of its ethics review board. Before participating in the NHANES, all participants provided written informed consent[17,18]. NHANES data can be accessed by the public through the NHANES website (http://www. cdc. gov/nchs/nhanes. htm). Of the 31,126 individuals who participated in the in-home interview, we excluded those aged below 40 years (n=21,156) because an ankle brachial index (ABI) was not measured in this age group. Participants were excluded from the study if they had missing bilateral ABI data (n=2408),ABI values exceeding 1.4 (n=65) or were lacking diet data (n=169). Individuals with missing covariates were excluded (n=740), resulting in a final analysis sample of 6,588 participants.

Ankle-brachial index

Systolic blood pressure measurements were taken from the right arm (brachial artery) and both ankles (posterior tibial artery) after a brief period of rest. If the participant's right arm measurements were unavailable due to any conditions that could affect accuracy, such as open wounds or dialysis shunts, the left arm was used for the brachial pressure measurement. Individuals aged 40-59 had their systolic blood pressure measured twice, while those aged 60 and older had it measured only once. The ankle-brachial index (ABI) was calculated by dividing the average systolic blood pressure (ASBP) of the ankle by the ASBP in the arm. For participants aged 60 and older, the single reading represented the mean values. ABI <0.9 was used to define PAD[19,20].

Dietary vitamin E intake

Data related to vitamin E intake in diets was gathered from the NHANES 1999 to 2004 first-day dietary interview examination files. A 24-hour dietary recall interview was conducted via the NHANES computer-assisted dietary data interview system to acquire detailed dietary intake data for all participants. This interview captured details of all foods and beverages consumed within a 24-hour duration, such as consumption time, eating occasion, food descriptions, portion sizes, food sources, and location of consumption. Following the dietary recall, a set of health-related inquiries were made. The data collection approach utilized the Automated Multiple Pass Method, which is the dietary data collection tool of the US Department of Agriculture (USDA).Detailed methodologies for the dietary survey can be found in the NHANES Dietary Interviewer’s Procedure Manual[15]. While 24-h dietary recalls possess inherent limitations in terms of reliability and effectiveness, they offer a greater level of specificity regarding the varieties and amounts of ingested foods than food frequency questionnaires[21–23]. The participants were then divided into tertile (Q1–Q3) based on their dietary vitamin E consumption.

Covariates

Standardized questionnaires were administered to obtain socio-demographic and lifestyle information. The Mobile Examination Center furnished the results of the examinations, including

body mass index (BMI), blood pressure, and various biochemical parameters. Different potential covariates were evaluated using the available literature[24], including age, sex, race/ethnicity, education level, marital status, family income, smoking status, physical activity, BMI, laboratory parameters (total cholesterol and glycated hemoglobin A1c), and comorbidities (e.g., diabetes, hypertension, and cardiovascular disease [CVD]). For detailed information regarding these variables, such as the methods used for measurement, questionnaire data, and the list of variables, please refer to the official NHANES website (www.cdc.gov/nchs/nhanes/ accessed on 1 May 2022). The categorization of family income was based on the poverty income ratio (PIR) into three groups: low (PIR ≤ 1.3), medium (PIR > 1.3–3.5), and high (PIR > 3.5). Smoking status was classified according to established definitions from previous studies: never smoked, current smoker, and former smoker. To evaluate physical activity levels, participants were asked about intense exercises leading to notable changes in breathing and heart rate (such as swimming or high-speed cycling), as well as moderate exercises causing mild-to-moderate increases in these physiological parameters (for example, golfing or leisurely biking). The duration of each activity should have been at least 10 min within the last month. Physical activity levels were categorized into three groups: below-moderate (lacking both moderate and intense activities), moderate (absence of intense exercise but inclusion of at least one moderately active pursuit), and high-intensity (presence of at least one episode involving rigorous exertion).

The identification of previous illnesses (such as hypertension and diabetes) was established through a questionnaire by asking participants if they had previously informed a doctor about their medical conditions. The history of cardiovascular disease was determined based on individuals' self-reported experiences with congestive heart failure, coronary heart disease, angina pectoris, heart attack, and stroke. BMI was calculated using a conventional method that considers weight and height.

Statistical analysis

This study used a rigorous and comprehensive approach to assess the effects of dietary vitamin E intake on PAD. Analysis of the association between dietary vitamin E intake and PAD was conducted using robust statistical techniques, such as binary logistic regression models that provide odds ratios and 95% confidence intervals, consideration of confounders based on expert judgment, existing scientific literature, and identification of significant covariates in univariate analysis, indicating a thoughtful approach to controlling potential sources of bias. Additionally, the use of a subgroup analysis to examine the relationship between vitamin E intake and PAD within specific subgroups adds to these findings. The handling of missing data through listwise deletion, sensitivity analyses to assess the robustness of the findings, and the reporting and comparison of effect sizes and p-values from various association inference models further enhanced the credibility and reliability of the study's conclusions. Overall, the methodology and analytical approach contributed to the robustness and validity of the findings.

Statistical analyses were performed using R Statistical Software (Version 4.2.2, http://www.R-project.org, The R Foundation) and the Free Statistics Analysis Platform (Version 1.9, Beijing, China, http://www.clinicalscientists.cn/freestatistics). A detailed explanation of their functionalities ensured transparency and reproducibility of the analytical methods used in this study. Overall, the statistical analysis appears to be comprehensive, well executed, and supported by appropriate software tools, contributing to the strength of the study's findings.

Results

Characteristics of the study population

In total, 31,126 individuals participated in the in-home interviews. Among them, 21,156 participants aged < 40 years were excluded. Furthermore, participants who had missing bilateral ABI data (n=2408), ABI values exceeding 1.4 (n=65), or lacking diet information (n=169) were excluded. Participants with incomplete covariate data were excluded (n=740), resulting in a final sample size of 6,588 participants (Fig 1).

Fig 1. Research flowchart

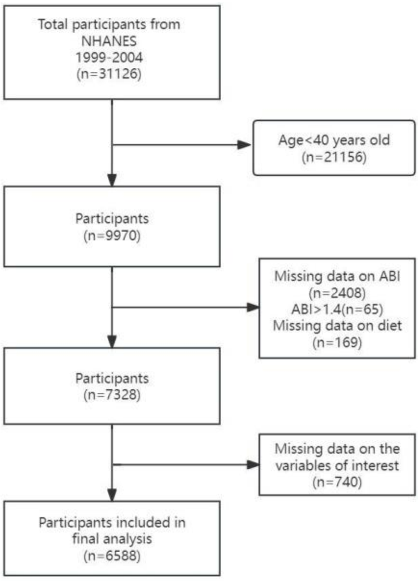

The study included qualified individuals aged 40 years or older with a PAD prevalence rate of 5.9%. Table 1 presents the basic features of the participants categorized according to their dietary consumption of vitamin E. The three groups differed in terms of sex, age, education level, race, MS, PIR, physical activity, HbA1c, smoking status, hypertension, CVD, and diabetes mellitus (P < 0.05). However, the distribution of patient characteristics, such as BMI and total cholesterol, among the dietary vitamin E intake groups was similar (P > 0.05)(Table 1).

Table 1. Baseline characteristics stratified by the dietary vitamin E intake quartiles (Q).

Variables	Total (n = 6588)	Q1 (n = 2191)	Q2 (n = 2195)	Q3 (n = 2202)	*p-* Value
**Gender, n (%)**					< 0.001
Male	3362 (51.0)	951 (43.4)	1086 (49.5)	1325 (60.2)	
Female	3226 (49.0)	1240 (56.6)	1109 (50.5)	877 (39.8)	
**Age (years)**	60.1 ± 13.0	61.6 ± 12.9	60.2 ± 13.2	58.4 ± 12.6	< 0.001
Race, n (%)					< 0.001

Mexican American	1371 (20.8)	507 (23.1)	449 (20.5)	415 (18.8)	
Other Hispanic	261 (4.0)	93 (4.2)	84 (3.8)	84 (3.8)	
Non-Hispanic white	3635 (55.2)	1070 (48.8)	1238 (56.4)	1327 (60.3)	
Non-Hispanic black	1127 (17.1)	453 (20.7)	355 (16.2)	319 (14.5)	
Other races	194 (2.9)	68 (3.1)	69 (3.1)	57 (2.6)	
**Education level,, n (%)**					< 0.001
Less than high school	1140 (17.3)	513 (23.4)	352 (16)	275 (12.5)	
High school diploma or GED	2562 (38.9)	908 (41.4)	854 (38.9)	800 (36.3)	
More than high school	2886 (43.8)	770 (35.1)	989 (45.1)	1127 (51.2)	
**MS, n (%)**					< 0.001
Married/Living with partner	4381 (66.5)	1316 (60.1)	1476 (67.2)	1589 (72.2)	
Widowed/Divorced/Separated	1839 (27.9)	746 (34)	604 (27.5)	489 (22.2)	
Never married	368 (5.6)	129 (5.9)	115 (5.2)	124 (5.6)	
**PIR, n (%)**					< 0.001
Low income	1504 (22.8)	635 (29)	490 (22.3)	379 (17.2)	
Medium income	2290 (34.8)	785 (35.8)	764 (34.8)	741 (33.7)	
High income	2794 (42.4)	771 (35.2)	941 (42.9)	1082 (49.1)	
**Physical activity, n (%)**					< 0.001
Sedentary	3017 (45.8)	1190 (54.3)	989 (45.1)	838 (38.1)	
Moderate	2071 (31.4)	629 (28.7)	706 (32.2)	736 (33.4)	
Vigorous	1500 (22.8)	372 (17)	500 (22.8)	628 (28.5)	
**BMI, kg/m2**	28.5 ± 5.6	28.3 ± 5.5	28.4 ± 5.6	28.7 ± 5.6	0.135
**Total cholesterol, mg/dL**	209.6 ± 41.3	210.9 ± 41.9	209.0 ± 41.0	208.7 ± 41.0	0.150
**HbA1c, %**	5.8 ± 1.1	5.8 ± 1.2	5.8 ± 1.0	5.7 ± 1.1	0.003
**Diabetes, n (%)**					
No	5742 (87.2)	1864 (85.1)	1921 (87.5)	1957 (88.9)	
Yes	846 (12,8)	327 (14.9)	274 (12,5)	245 (11.1)	
**Hypertension, n (%)**					0.012
No	4251 (64.5)	1361 (62.1)	1432 (65.2)	1458 (66.2)	
Yes	2337 (35.5)	830 (37.9)	763 (34.8)	744 (33.8)	
**CVD, n (%)**					0.025
No	5546 (84.2)	1809 (82.6)	1854 (84.5)	1883 (85.5)	
Yes	1042 (15.8)	382 (17.4)	341 (15.5)	319 (14.5)	
**Smoking, n (%)**					< 0.001
Never	3043 (46.2)	1011 (46.1)	1038 (47.3)	994 (45.1)	
Former	2288 (34.7)	707 (32.3)	753 (34.3)	828 (37.6)	
Current	1257 (19.1)	473 (21.6)	404 (18.4)	380 (17.3)	
**PAD, n (%)**					< 0.001
No	6199 (94.1)	2023 (92.3)	2054 (93.6)	2122 (96.4)	
Yes	389 (5.9)	168 (7.7)	141 (6.4)	80 (3.6)	

Values for BMI and age Values are given as mean ± standard deviation , other values are presented as numbers and percentages. BMI, body mass index; CVD, cardiovascular disease; CRP, C-reactive protein; HbA1c, glycosylated hemoglobin; MS, marital status; PIR, poverty income ratio; PAD, peripheral arterial disease.

Association between dietary vitamin E intake and PAD

In the multivariable logistic regression analyses, it was found that participants in the Q3 group exhibited a lower probability of PAD (OR=0.68, 95% CI=0.51–0.91, P=0.009) than the Q1 group, after adjusting for potential confounders (Table 2, model 5).

The adjusted smoothed plots suggested a straightforward linear relationship between dietary vitamin E intake and PAD (Figure 2; P for nonlinearity = 0.897; the highest and lowest 0.5% were trimmed for each dietary vitamin E intake measure). As the dietary vitamin E intake increased, the risk of PAD decreased.

Figure 2 shows the correlation between dietary vitamin E intake and PAD in both males and females. Data were analyzed using a multivariate logistic regression model with restricted cubic splines. To account for various factors, such as sex, age, BMI, severity of illness, and reason for admission (Model 5), dietary vitamin E intake was considered a continuous variable. The reference standard was the median dietary vitamin E intake. Shaded areas represent 95% confidence intervals. The curves highlight the inverse relationship between dietary vitamin E intake and PAD when considering low or near-median values of dietary vitamin E intake. Owing to limited patient numbers and the nature of the cubic fit analysis, there was significant variability in the 95% confidence intervals at extreme values.

Table 2. Odds ratios (ORs) and 95% confidence interval (CI) of the dietary vitaminE intake quartiles for PAD.

Variable	VitaminE(mg) OR (95%CI)	P-value	Q1 (0-4.4) OR (95%CI)	Q2 (4.41-7.69) OR (95%CI)	P-value	Q3 (7.7-97.0) OR (95%CI)	P-value	Trend.test	P-value
**Model 1**	0.944(0.92~0.969)	<0.001	1 (Ref)	0.83 (0.66~1.04)	0.108	0.45 (0.35~0.6)	<0.001	0.69 (0.61~0.79)	<0.001
**Model 2**	0.973 (0.949~0.997)	0.0279	1 (Ref)	0.97 (0.76~1.23)	0.779	0.63 (0.47~0.84)	0.002	0.81 (0.71~0.93)	0.003
**Model 3**	0.975 (0.951~0.999)	0.0406	1 (Ref)	0.97 (0.76~1.24)	0.808	0.64 (0.48~0.86)	0.003	0.82 (0.71~0.94)	0.005
**Model 4**	0.976 (0.952~1)	0.0517	1 (Ref)	0.97 (0.76~1.24)	0.804	0.65 (0.48~0.86)	0.003	0.82 (0.71~0.94)	0.005
**Model 5**	0.981 (0.957~1.004)	0.1075	1 (Ref)	1 (0.78~1.28)	0.992	0.68 (0.51~0.91)	0.009	0.84 (0.73~0.97)	0.016

Data are presented as odds ratios, 95% CIs (confidence intervals), and p-value.

Model 1 adjusted for none.

Model 2 adjusted for age,gender,race, education level,MS, physical activity.

Model 3 adjusted for age,gender,race, education level,MS, physical activity,PIR,BMI,total cholesterol, HbA1c.

Model 4 adjusted for age,gender,race, education level,MS, physical activity,PIR,BMI,total cholesterol,HbA1c, diabetes,hypertension,CVD.

Model 5 adjusted for all covariates.

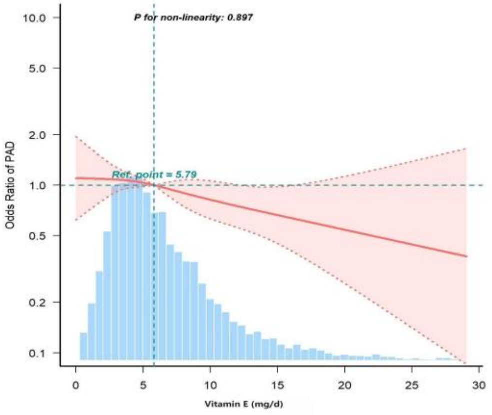

Fig2.Association between dietary vitamin E intake and PAD odds ratio. Solid and dashed lines represent the predicted value and 95% confidence intervals. They were adjusted for age, sex, race/ethnicity, education level, marital status, family income, smoking status, physical activity, BMI, total cholesterol, glycated hemoglobin A1c, and comorbidities. Only 99% of the date is shown.

Stratification analysis

In this study, we performed a subgroup analysis based on various factors, including age, sex, ethnicity, presence of hypertension or diabetes, educational background, history of CVD, income level (PIR), physical activity level, and smoking status. Additionally, sensitivity and supplementary analyses were conducted, and the results of the subgroup analysis indicated no significant evidence of effect modification or interaction based on common risk factors for PAD (all P values for interaction were >0.05) (Fig 3).

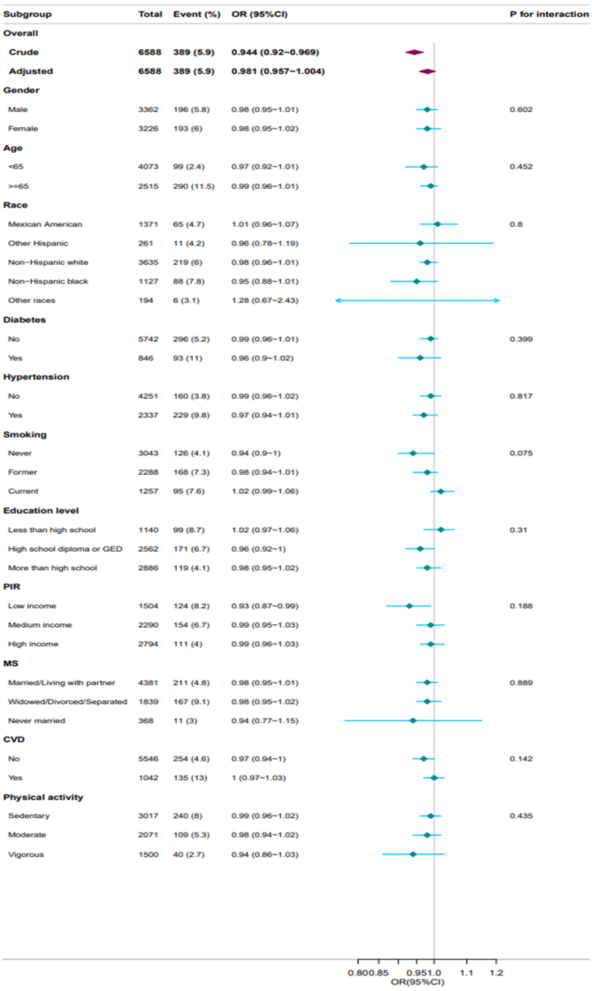

Fig3.The relationship between dietary vitamin E intake and PAD according to basic features. Except for the stratification component itself, each stratification factor was adjusted for all other variables.

Discussion

To the extent of our understanding, in a retrospective cross-sectional study conducted on a significant number of participants involved in the NHANES between 1999 and 2004 in the United States, we consistently observed an inverse correlation between the intake of dietary vitamin E and likelihood of developing PAD. Results were consistent across clinical subgroups and in sensitivity analyses. These observations would have important implications for current PAD management strategies.

Vitamin E, a membrane-bound antioxidant found in nuts, vegetable oils, and fish, is associated with reduced cellular damage in cardiovascular disease[21]. Its antioxidant and anti-inflammatory properties, along with its ability to prevent platelet aggregation and smooth cell proliferation, have been shown to exhibit effects that counteract the development of atherosclerosis [25].
Our research findings align with previous observational studies indicating an inverse relationship between the dietary intake of vitamin E and the occurrence of PAD among participants in the NHANES conducted from 1999 to 2004[9,14,16]. According to a study conducted by Lane et al.[21], an increased intake of vitamin E exhibited a notable safeguarding effect, regardless of the presence of conventional cardiovascular risk factors, within the population of the United States. Not come singly but in pairs, Antonelli-Incalzi et al.[26] conducted a study on the nutrient consumption of 1251 individuals living in their own homes, who were part of the InCHIANTI research. The average age of participants were 68 years. They also investigated whether there was a potential decrease in the risk of PAD associated with a daily intake of Vitamin E equal to or greater than 7.726 mg/d. (OR: 0.37; 95% CI 0.16–0.84). In addition, the Rotterdam Study found that there was a negative correlation between vitamin E consumption and PAD in men. However, it remains unclear whether the association between antioxidant intake and PAD differs between sexes or whether these findings are influenced by the different dietary patterns observed in men than in women [27]. Further research is necessary to validate our results and investigate the intricate relationships and potential mechanisms involved.
Our findings support and expand upon previous research conducted on participants in the NHANES from 1999 to 2004 in the United States. We found that a higher intake of dietary vitamin E was associated with a reduced risk of PAD after accounting for all relevant factors (OR, 0.981; 95% CI, 0.957–1.004). When we divided dietary vitamin E intake into quartiles, individuals in the third quartile had a lower incidence rate of PAD than those in the first quartile (OR, 0.68; 95% CI: 0.51, 0.91). Subgroup analysis yielded similar results without any significant differences (all P values for interactions were >0.05). Extensive clinical research has demonstrated the potential protective effects of vitamin E against long-term cardiovascular diseases[28]. Studies have indicated that taking vitamin E supplements for more than four years can result in a substantial 59% decrease in mortality from coronary disease[29]. The discussion regarding the impact of vitamin E on cardiovascular diseases is complex. While certain studies indicate that vitamin E supplements may provide protection against cardiovascular diseases, others do not support this claim. Additionally, a minority of studies have even suggested potential adverse effects[30,31]. So further investigations are necessary to validate our findings and explore the intricate relationships and potential mechanisms involved.

This was a well-designed retrospective cross-sectional study with a robust sample size and stringent inclusion and exclusion criteria. Baseline data on a wide range of lifestyle factors, including dietary cofactors, were collected, enabling adjustment for potential confounders in the analyses. Additionally, a comprehensive set of sensitivity analyses was conducted to enhance the interpretation of the results and validate the main findings.

However, this study had several limitations. First, owing to the cross-sectional and observational nature of the analysis, causal relationships among dietary vitamin E intake, covariates, and PAD could not be definitively established. Despite efforts to control for relevant confounders in the multivariate model, the presence of unmeasured or unknown residual confounders, such as dietary habits and family income, may have led to an overestimation of the observed associations. Furthermore, in this study, the intake of vitamin E was not energy-adjusted.Nonetheless, the study mainly included American adults, which limits the generalizability of the findings and necessitates further research on diverse populations.

Conclusions

Our study provides supporting evidence indicating a detrimental correlation between the consumption of dietary vitamin E and the occurrence of PAD in US adults older than 40 years. Therefore, individuals with insufficient dietary vitamin E intake, especially those with a very low vitamin E intake, should consider increasing their vitamin E intake to lower the risk of developing PAD. These findings should be considered when offering dietary guidance to prevent PAD .

Statements

Acknowledgement

We express our gratitude to Jie Liu from the Department of Vascular and Endovascular Surgery at Chinese PLA General Hospital for providing valuable assistance in statistical analysis, study design consultations, and offering insightful feedback on the manuscript.

Statement of Ethics

Study approval statement: The study did not require any additional institutional review board approval for the secondary analysis, thus ethical review and approval were exempted. Consent to participate statement: The NHANES study was approved by the Ethics Review Committee of the National Center for Health Statistics (NCHS), and prior to their involvement, all participants provided written consent after being fully informed.

Conflict of Interest Statement

The authors have no conflicts of interest to declare.

Funding Sources

This study was not financially supported by any funding agencies in the public, commercial, or non-profit sectors.

Author Contributions

Conceptualization, Qiang Liu and Jianjun Shi; Data curation, Qiang Liu and Xing Wu; Formal analysis and Methodology, Qiang Liu, Xing Wu and Jianjun Shi; Funding acquisition, Yun Wang and Jianjun Shi; Writing—original draft, Qiang Liu and Xing Wu; Writing—Xiang Wang and Fei Zhao; All the authors have carefully reviewed and given their consent to the final version of the manuscript.

Data Availability Statement

All NHANES data and information are publicly available at https://www.cdc.gov/nchs/nhanes/.~~Publicly accessible datasets for this study can be found online. The name of the repository/repositories and their corresponding accession numbers are provided at http://www.cdc.gov/nchs/nhanes/ (accessed on 1 May 2022).~~

References

Formatted: Font:

Formatted: Font:

Formatted: Not Expanded by / Condensed by

Formatted: Not Expanded by / Condensed by

Formatted: Not Expanded by / Condensed by

Formatted: Not Expanded by / Condensed by

Formatted: Not Expanded by / Condensed by

Formatted: Not Expanded by / Condensed by

Formatted: Not Expanded by / Condensed by

Formatted: Not Expanded by / Condensed by

Formatted: Not Expanded by / Condensed by

Formatted: Not Expanded by / Condensed by

Formatted: Not Expanded by / Condensed by

Formatted: Not Expanded by / Condensed by

Formatted: Not Expanded by / Condensed by

Formatted: Not Expanded by / Condensed by

Formatted: Not Expanded by / Condensed by

Formatted: Not Expanded by / Condensed by

Formatted: Not Expanded by / Condensed by

Formatted: Not Expanded by / Condensed by

Formatted: Not Expanded by / Condensed by

Formatted: Not Expanded by / Condensed by

Formatted: Not Expanded by / Condensed by

Formatted: Font: (Default) +Body (Calibri), 12 pt

Formatted: Not Expanded by / Condensed by

Formatted: Not Expanded by / Condensed by

Formatted: Not Expanded by / Condensed by

Formatted: Not Expanded by / Condensed by

Formatted: Not Expanded by / Condensed by

Formatted: Font: (Default) +Body (Calibri), 12 pt, Not Expanded by / Condensed by

1. Poledniczek M, Neumayer C, Kopp CW, Schlager O, Gremmel T, Jozkowicz A, et al. Micro- and Macrovascular Effects of Inflammation in Peripheral Artery Disease—Pathophysiology and Translational Therapeutic Approaches. Biomedicines. 2023;11:2284.

2. Zhang Z, Chen Z. Higher Systemic Immune-Inflammation Index is Associated With Higher Likelihood of Peripheral Arterial Disease. Ann Vasc Surg. 2022;84:322–6.

3. Hirsch AT, Haskal ZJ, Hertzer NR, Bakal CW, Creager MA, Halperin JL, et al. ACC/AHA 2005 Guidelines for the Management of Patients With Peripheral Arterial Disease (Lower Extremity, Renal, Mesenteric, and Abdominal Aortic): Executive Summary A Collaborative Report From the American Association for Vascular Surgery/Society for Vascular Surgery,∗∗AAVS/SVS when Guideline initiated, now merged into SVS Society for Cardiovascular Angiography and Interventions, Society for Vascular Medicine and Biology, Society of Interventional Radiology, and the ACC/AHA Task Force on Practice Guidelines (Writing Committee to Develop Guidelines for the Management of Patients With Peripheral Arterial Disease). Journal of the American College of Cardiology. 2006;47:1239–312.

4. Correction to: 2024 Heart Disease and Stroke Statistics: A Report of US and Global Data From the American Heart Association. Circulation. 2024;149:e1164.

5. Zhu K, Qian F, Lu Q, Li R, Qiu Z, Li L, et al. Modifiable Lifestyle Factors, Genetic Risk, and Incident Peripheral Artery Disease Among Individuals With Type 2 Diabetes: A Prospective Study. Diabetes Care. 2024;47:435–43.

6. Pan D, Guo J, Su Z, Meng W, Wang J, Guo J, et al. Association of prognostic nutritional index with peripheral artery disease in US adults: a cross-sectional study. BMC Cardiovasc Disord. 2024;24:133.

7. Joosten MM, Pai JK, Bertoia ML, Rimm EB, Spiegelman D, Mittleman MA, et al. Associations between conventional cardiovascular risk factors and risk of peripheral artery disease in men. JAMA. 2012;308:1660–7.

8. Navab M, Ananthramaiah GM, Reddy ST, Van Lenten BJ, Ansell BJ, Fonarow GC, et al. The oxidation hypothesis of atherogenesis: the role of oxidized phospholipids and HDL. J Lipid Res. 2004;45:993–1007.

9. Naqvi AZ, Davis RB, Mukamal KJ. Nutrient intake and peripheral artery disease in adults: key considerations in cross-sectional studies. Clin Nutr. 2014;33:443–7.

10. Min J-Y, Cho J-S, Lee K-J, Park J-B, Park S-G, Kim JY, et al. Potential role for organochlorine pesticides in the prevalence of peripheral arterial diseases in obese persons: results from the National Health and Nutrition Examination Survey 1999-2004. Atherosclerosis. 2011;218:200–6.

11. Selvin E, Köttgen A, Coresh J. Kidney function estimated from serum creatinine and cystatin C and peripheral arterial disease in NHANES 1999-2002. Eur Heart J. 2009;30:1918–25.

12. Colombo ML. An update on vitamin E, tocopherol and tocotrienol-perspectives. Molecules. 2010;15:2103–13.

13. Gahche J, Bailey R, Burt V, Hughes J, Yetley E, Dwyer J, et al. Dietary supplement use among U.S. adults has increased since NHANES III (1988-1994). NCHS Data Brief. 2011;1–8.

14. Wu Z, Ruan Z, Liang G, Wang X, Wu J, Wang B. Association between dietary magnesium intake and peripheral arterial disease: Results from NHANES 1999-2004. PLoS One. 2023;18:e0289973.

15. Hicks CW, Wang D, Matsushita K, McEvoy JW, Christenson R, Selvin E. Glycated albumin and HbA1c as markers of lower extremity disease inUS adults with and without diabetes. Diabetes Res Clin Pract. 2022;184:109212.

16. Qu C-J, Teng L-Q, Liu X-N, Zhang Y-B, Fang J, Shen C-Y. Dose-Response Relationship Between Physical Activity and the Incidence of Peripheral Artery Disease in General Population: Insights From the National Health and Nutrition Examination Survey 1999-2004. Front Cardiovasc Med. 2021;8:730508.

17. Fan H, Zhou J, Huang Y, Feng X, Dang P, Li G, et al. A Proinflammatory Diet Is Associated with Higher Risk of Peripheral Artery Disease. Nutrients. 2022;14.

18. Liu Y, Chang L, Wu M, Xu B, Kang L. Triglyceride Glucose Index Was Associated With the Risk of Peripheral Artery Disease. Angiology. 2022;73:655–9.

19. Loreaux F, Jéhannin P, Le Pabic E, Paillard F, Le Faucheur A, Mahe G. An unfavorable dietary pattern is associated with symptomatic peripheral artery disease. Nutr Metab Cardiovasc Dis. 2024;34:2173–81.

20. Liu Y, Wei R, Tan Z, Chen G, Xu T, Liu Z, et al. Association between dietary fiber intake and peripheral artery disease in hypertensive patients. J Health Popul Nutr. 2024;43:118.

21. Lane JS, Magno CP, Lane KT, Chan T, Hoyt DB, Greenfield S. Nutrition impacts the prevalence of peripheral arterial disease in the United States. Journal of Vascular Surgery. 2008;48:897-904.e1.

22. Mazidi M, Mikhailidis DP, Banach M. Higher dietary acid load is associated with higher likelihood of peripheral arterial disease among American adults. J Diabetes Complications. 2018;32:565–9.

23. Mattioli AV, Francesca C, Mario M, Alberto F. Fruit and vegetables in hypertensive women with asymptomatic peripheral arterial disease. Clin Nutr ESPEN. 2018;27:110–2.

24. Cheng Y, Fang Z, Zhang X, Wen Y, Lu J, He S, et al. Association between triglyceride glucose-body mass index and cardiovascular outcomes in patients undergoing percutaneous coronary intervention: a retrospective study. Cardiovasc Diabetol. 2023;22:75.

25. Kumar M, Deshmukh P, Kumar M, Bhatt A, Sinha AH, Chawla P. Vitamin E Supplementation and Cardiovascular Health: A Comprehensive Review. Cureus [Internet]. 2023 [cited 2024 Mar 10]; Available from: https://www.cureus.com/articles/184881-vitamin-e-supplementation-and-cardiovascular-health-a-comprehensive-review.

26. Antonelli-Incalzi R, Pedone C, McDermott MM, Bandinelli S, Miniati B, Lova RM, et al. Association between nutrient intake and peripheral artery disease: Results from the InCHIANTI study. Atherosclerosis. 2006;186:200–6.

27. Klipstein-Grobusch K. Dietary Antioxidants and Peripheral Arterial Disease : The Rotterdam Study. American Journal of Epidemiology. 2001;154:145–9.

28. Azzi A, Meydani SN, Meydani M, Zingg JM: The rise, the fall and the renaissance of vitamin E . Arch Biochem Biophys. 2016, 595:100-8.

29. Muntwyler J, Hennekens CH, Manson JE, Buring JE, Gaziano JM: Vitamin supplement use in a low-risk population of US male physicians and subsequent cardiovascular mortality . Arch Intern Med. 2002, 162:1472-6.

30. Mezzetti A, Zuliani G, Romano F, Costantini F, Pierdomenico SD, Cuccurullo F, Fellin R:

Vitamin E and lipid peroxide plasma levels predict the risk of cardiovascular events in a group of healthy very old people . J Am Geriatr Soc. 2001, 49:533-7.

31. Wright ME, Lawson KA, Weinstein SJ, Pietinen P, Taylor PR, Virtamo J, Albanes D: Higher baseline serum concentrations of vitamin E are associated with lower total and cause-specific mortality in the AlphaTocopherol, Beta-Carotene Cancer Prevention Study . Am J Clin Nutr. 2006, 84:1200-7.

Figure 图 Click here to access/download;Figure;figure and table.docx

Fig 1. Research flowchart

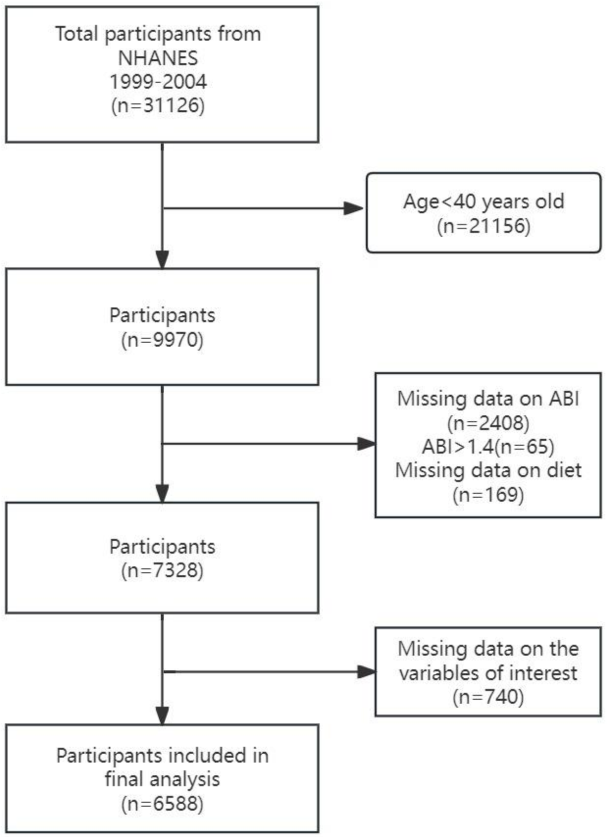

Table 1. Baseline characteristics stratified by the dietary vitaminE intake quartiles (Q).

Variables	Total (n = 6588)	Q1 (n = 2191)	Q2 (n = 2195)	Q3 (n = 2202)	*p*- Value
**Gender, n (%)**					< 0.001
Male	3362 (51.0)	951 (43.4)	1086 (49.5)	1325 (60.2)	
Female	3226 (49.0)	1240 (56.6)	1109 (50.5)	877 (39.8)	
**Age (years)**	60.1 ± 13.0	61.6 ± 12.9	60.2 ± 13.2	58.4 ± 12.6	< 0.001
Race, n (%)					< 0.001
Mexican American	1371 (20.8)	507 (23.1)	449 (20.5)	415 (18.8)	
Other Hispanic	261 (4.0)	93 (4.2)	84 (3.8)	84 (3.8)	
Non-Hispanic white	3635 (55.2)	1070 (48.8)	1238 (56.4)	1327 (60.3)	
Non-Hispanic black	1127 (17.1)	453 (20.7)	355 (16.2)	319 (14.5)	
Other races	194 (2.9)	68 (3.1)	69 (3.1)	57 (2.6)	
**Education level,, n (%)**					< 0.001
Less than high school	1140 (17.3)	513 (23.4)	352 (16)	275 (12.5)	
High school diploma or GED	2562 (38.9)	908 (41.4)	854 (38.9)	800 (36.3)	
More than high school	2886 (43.8)	770 (35.1)	989 (45.1)	1127 (51.2)	
**MS, n (%)**					< 0.001
Married/Living with partner	4381 (66.5)	1316 (60.1)	1476 (67.2)	1589 (72.2)	
Widowed/Divorced/Separated	1839 (27.9)	746 (34)	604 (27.5)	489 (22.2)	
Never married	368 (5.6)	129 (5.9)	115 (5.2)	124 (5.6)	
**PIR, n (%)**					< 0.001
Low income	1504 (22.8)	635 (29)	490 (22.3)	379 (17.2)	
Medium income	2290 (34.8)	785 (35.8)	764 (34.8)	741 (33.7)	
High income	2794 (42.4)	771 (35.2)	941 (42.9)	1082 (49.1)	
**Physical activity, n (%)**					< 0.001
Sedentary	3017 (45.8)	1190 (54.3)	989 (45.1)	838 (38.1)	
Moderate	2071 (31.4)	629 (28.7)	706 (32.2)	736 (33.4)	
Vigorous	1500 (22.8)	372 (17)	500 (22.8)	628 (28.5)	
**BMI, kg/m2**	28.5 ± 5.6	28.3 ± 5.5	28.4 ± 5.6	28.7 ± 5.6	0.135
**Total cholesterol, mg/dL**	209.6 ± 41.3	210.9 ± 41.9	209.0 ± 41.0	208.7 ± 41.0	0.150
**HbA1c, %**	5.8 ± 1.1	5.8 ± 1.2	5.8 ± 1.0	5.7 ± 1.1	0.003

**Diabetes, n (%)**					
No	5742 (87.2)	1864 (85.1)	1921 (87.5)	1957 (88.9)	
Yes	846 (12,8)	327 (14.9)	274 (12,5)	245 (11.1)	
**Hypertension, n (%)**					0.012
No	4251 (64.5)	1361 (62.1)	1432 (65.2)	1458 (66.2)	
Yes	2337 (35.5)	830 (37.9)	763 (34.8)	744 (33.8)	
**CVD, n (%)**					0.025
No	5546 (84.2)	1809 (82.6)	1854 (84.5)	1883 (85.5)	
Yes	1042 (15.8)	382 (17.4)	341 (15.5)	319 (14.5)	
**Smoking, n (%)**					< 0.001
Never	3043 (46.2)	1011 (46.1)	1038 (47.3)	994 (45.1)	
Former	2288 (34.7)	707 (32.3)	753 (34.3)	828 (37.6)	
Current	1257 (19.1)	473 (21.6)	404 (18.4)	380 (17.3)	
**PAD, n (%)**					< 0.001
No	6199 (94.1)	2023 (92.3)	2054 (93.6)	2122 (96.4)	
Yes	389 (5.9)	168 (7.7)	141 (6.4)	80 (3.6)	

Values are given as mean ± standard deviation or numbers and percentages. BMI, body mass index; CVD, cardiovascular disease; CRP, C-reactive protein; HbA1c, glycosylated hemoglobin; MS, marital status; PIR, poverty income ratio; PAD, peripheral arterial disease.

Table 2. Odds ratios (ORs) and 95% confidence interval (CI) of the dietary vitaminE intake quartiles for PAD.

Variable	VitaminE(mg) OR (95%CI)	P-value	Q1 (0-4.4) OR (95%CI)	Q2 (4.41-7.69) OR (95%CI)	P-value	Q3 (7.7-97.0) OR (95%CI)	P-value	Trend.test	P-value
**Model 1**	0.944(0.92~0.969)	<0.001	1 (Ref)	0.83 (0.66~1.04)	0.108	0.45 (0.35~0.6)	<0.001	0.69 (0.61~0.79)	<0.001
**Model 2**	0.973 (0.949~0.997)	0.0279	1 (Ref)	0.97 (0.76~1.23)	0.779	0.63 (0.47~0.84)	0.002	0.81 (0.71~0.93)	0.003
**Model 3**	0.975 (0.951~0.999)	0.0406	1 (Ref)	0.97 (0.76~1.24)	0.808	0.64 (0.48~0.86)	0.003	0.82 (0.71~0.94)	0.005
**Model 4**	0.976 (0.952~1)	0.0517	1 (Ref)	0.97 (0.76~1.24)	0.804	0.65 (0.48~0.86)	0.003	0.82 (0.71~0.94)	0.005
**Model 5**	0.981 (0.957~1.004)	0.1075	1 (Ref)	1 (0.78~1.28)	0.992	0.68 (0.51~0.91)	0.009	0.84 (0.73~0.97)	0.016

Data are presented as odds ratios, 95% CIs (confidence intervals), and p-value.

Model 1 adjusted for none.

Model 2 adjusted for age,gender,race, education level,MS, physical activity.

Model 3 adjusted for age,gender,race, education level,MS, physical activity,PIR,BMI,total cholesterol, HbA1c.

Model 4 adjusted for age,gender,race, education level,MS, physical activity,PIR,BMI,total cholesterol,HbA1c, diabetes,hypertension,CVD.

Model 5 adjusted for all covariates.

Fig 2.

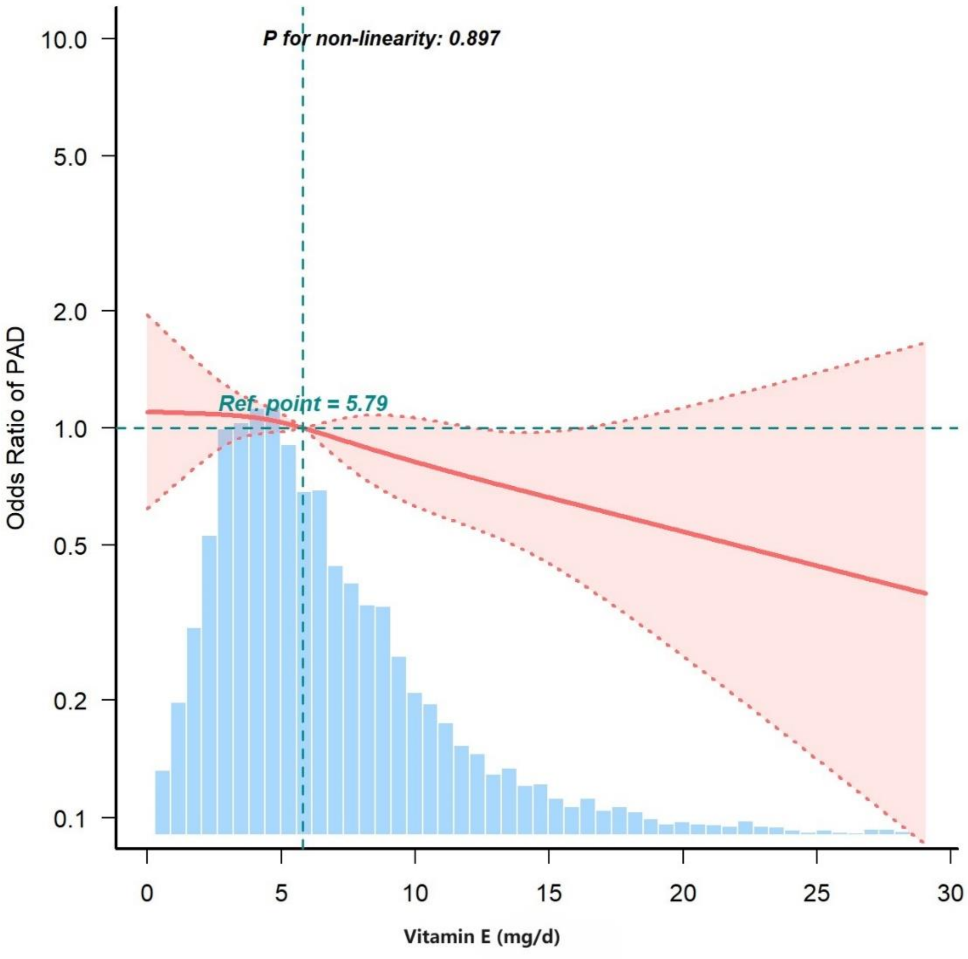
P for non-linearity: 0.897
Ref. point = 5.79
Odds Ratio of PAD
10.0
5.0
2.0
1.0
0.5
0.2
0.1
0
5
10
15
20
25
30
Vitamin E (mg/d)

Fig 3.

Subgroup	Total	Event (%)	OR (95%CI)	P for interaction
**Overall**				
**Crude**	**6588**	**389 (5.9)**	**0.944 (0.92~0.969)**	
**Adjusted**	**6588**	**389 (5.9)**	**0.981 (0.957~1.004)**	
**Gender**				
Male	3362	196 (5.8)	0.98 (0.95~1.01)	0.602
Female	3226	193 (6)	0.98 (0.95~1.02)	
**Age**				
<65	4073	99 (2.4)	0.97 (0.92~1.01)	0.452
>=65	2515	290 (11.5)	0.99 (0.96~1.01)	
**Race**				
Mexican American	1371	65 (4.7)	1.01 (0.96~1.07)	0.8
Other Hispanic	261	11 (4.2)	0.96 (0.78~1.19)	
Non−Hispanic white	3635	219 (6)	0.98 (0.96~1.01)	
Non−Hispanic black	1127	88 (7.8)	0.95 (0.88~1.01)	
Other races	194	6 (3.1)	1.28 (0.67~2.43)	
**Diabetes**				
No	5742	296 (5.2)	0.99 (0.96~1.01)	0.399
Yes	846	93 (11)	0.96 (0.9~1.02)	
**Hypertension**				
No	4251	160 (3.8)	0.99 (0.96~1.02)	0.817
Yes	2337	229 (9.8)	0.97 (0.94~1.01)	
**Smoking**				
Never	3043	126 (4.1)	0.94 (0.9~1)	0.075
Former	2288	168 (7.3)	0.98 (0.94~1.01)	
Current	1257	95 (7.6)	1.02 (0.99~1.06)	
**Education level**				
Less than high school	1140	99 (8.7)	1.02 (0.97~1.06)	0.31
High school diploma or GED	2562	171 (6.7)	0.96 (0.92~1)	
More than high school	2886	119 (4.1)	0.98 (0.95~1.02)	
**PIR**				
Low income	1504	124 (8.2)	0.93 (0.87~0.99)	0.188
Medium income	2290	154 (6.7)	0.99 (0.95~1.03)	
High income	2794	111 (4)	0.99 (0.96~1.03)	
**MS**				
Married/Living with partner	4381	211 (4.8)	0.98 (0.95~1.01)	0.889
Widowed/Divorced/Separated	1839	167 (9.1)	0.98 (0.95~1.02)	
Never married	368	11 (3)	0.94 (0.77~1.15)	
**CVD**				
No	5546	254 (4.6)	0.97 (0.94~1)	0.142
Yes	1042	135 (13)	1 (0.97~1.03)	
**Physical activity**				
Sedentary	3017	240 (8)	0.99 (0.96~1.02)	0.435
Moderate	2071	109 (5.3)	0.98 (0.94~1.02)	
Vigorous	1500	40 (2.7)	0.94 (0.86~1.03)	

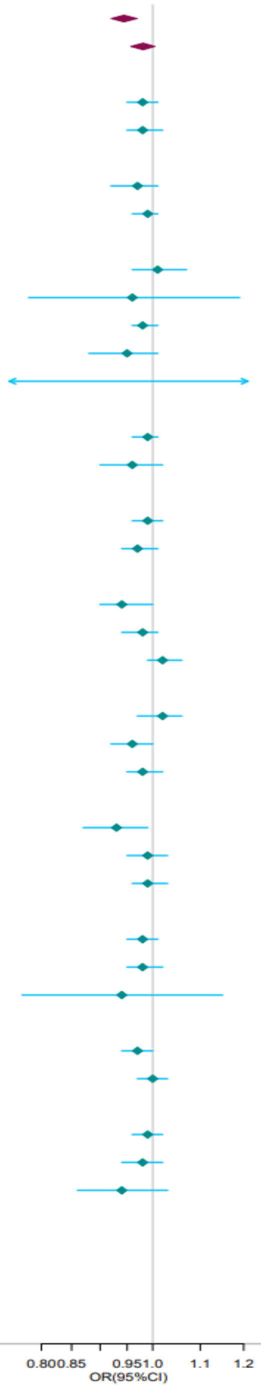

Research Article

Association of dietary vitamin E intake with peripheral arterial disease: A retrospective cross-sectional study

Qiang Liu[a,b,1], Xing Wu[c,d,1], Yun Wang[a,b], Xiang Wang[a,b], Fei Zhao[a,b], Jianjun Shi[a,b,*]

[a] Department of Cardiovascular Surgery, Taiyuan Central Hospital,China

[b] Department of Cardiovascular Surgery, the Ninth Clinical College Affiliated to Shanxi Medical University, China

[c] Department of Nephrology, Taiyuan Central Hospital, China

[d] Department of Nephrology, the Ninth Clinical College Affiliated to Shanxi Medical University, China

* Corresponding author

E-mail address: 13934282361@163.com

Keywords: dietary vitamin E intake; peripheral arterial disease; ankle-brachial index; National Health and Nutrition Examination Survey; cross-sectional study

Abstract

Background~~Introduction~~: The relationship between the amount of dietary vitamin E consumed and the development of peripheral arterial disease (PAD) remains a topic of debate. This relationship is of the utmost importance in the realms of healthcare and public health and is currently a highly researched and prominent topic.This study aimed to present~~gain~~ ~~deeper insights into~~ the relationship between dietary vitamin E intake and PAD.

Commented [A1]: You should use “present” instead of “gain”

Commented [A2R1]: Thank you, it has been modified as required

Methods: In a retrospective cross-sectional analysis, data from 6,588 participants in the National Health and Nutrition Examination Survey of the United States were examined during the period 1999–2004. We collected data on age, sex, race, marital status, education, physical activity, income, smoking, hypertension, diabetes, cardiovascular disease, body mass index, total cholesterol and HbA1c. Logistic regression and smooth curve fitting were used to support the research objectives.

Results: After accounting for all relevant factors, a negative correlation between dietary vitamin E intake and the likelihood of PAD was observed (OR: 0.981, 95% CI: 0.957–1.004). The overall prevalence of PAD was 5.9%, with 49.6% in males and 50.4% in females. Individuals in the third quartile of dietary vitamin E intake had a lower occurrence rate of peripheral artery disease than those in the first quartile (OR: 0.68, 95% CI: 0.51, 0.91). Similar patterns of association were observed in the subgroup analysis (all P values for interaction were >0.05).

Conclusions: Our study suggests a negative association between dietary vitamin E intake and incidence of PAD. Therefore individuals with insufficient dietary vitamin E intake, especially those with a very low intake, should consider increasing their vitamin E intake to lower the risk of developing PAD. These findings should be considered when offering dietary guidance to prevent PAD .

Introduction

Peripheral arterial disease (PAD) is caused by atherosclerosis, a condition characterized by the accumulation of fatty deposits in the arteries of the lower extremities and feet[1]. It is ~~a~~ prevalent disorder affecting ~~,~~over 200 million individuals worldwide [2]. In the United States, PAD affects around 8.5 million adults aged 40 years and above, with similar occurrence rates among men and women [3,4]. The economic and health burdens of PAD are substantial, emphasizing the need to identify its causes and implement appropriate measures. Previous studies have highlighted the significant roles of inflammatory markers, endothelial dysfunction, and oxidative stress in PAD development[5,6]. Although smoking, diabetes, hypertension, and dyslipidemia are established risk factors for PAD[3,7], the influence of diet is unclear. Further investigations are warranted to enhance our understanding of preventive measures and management strategies for PAD, given the limited knowledge of effective preventive factors.

Commented [A3]: The 'a' is redundant.

Commented [A4R3]: Thank you, it has been modified as required.

Commented [A5]: Space

Commented [A6R5]: Thank you, it has been modified as required.

Vitamin E, an antioxidant that dissolves in fat, is recognized as a key player in reducing lipid peroxidation in experimental settings[8]. Gey [4] first proposed the idea of vitamin E's potential in preventing cardiovascular disease in the "antioxidant hypothesis of atherosclerosis". Subsequent evidence linked vitamin E to atherosclerosis and thrombotic complications, attributing its anti-atherosclerotic activity to a range of biological functions, including antioxidant functions as scavengers of free radicals, in addition to non-antioxidant roles, such as controlling signal transduction, cellular proliferation, and gene expression. Vitamin E has attracted interest because of its anti-atherogenic and antioxidant properties, which have been associated with improved cardiovascular health in numerous studies. There was a significant increase in alpha-tocopherol levels [9–11].However, w~~w~~hile some studies indicate that supplemental vitamin E may offer protection against cardiovascular diseases, others do not, and a few even suggest the potential for adverse effects[12,13].

Commented [A7]: You should cite this author after his/her name.

Commented [A8R7]: Thank you, it has been modified as required.

Commented [A9]: Please check your grammar. Because there is no comma after 'however'.

Commented [A10R9]: Thank you, it has been modified as required.

Therefore, to address this knowledge gap, the ultimate goal was to present ~~gain~~ a deeper understanding of how dietary vitamin E intake influences PAD and the implications of this relationship. We sought to gain deeper insights into the relationship between dietary vitamin E intake and PAD through a retrospective cross-sectional study involving 6,588 participants in the National Health and Nutrition Examination Survey (NHANES). We aimed to provide valuable information for personalized nutritional management strategies and contribute to a deeper understanding of the nutritional status of individuals and populations.

Commented [A11]: You should use "present" instead of "gain"

Commented [A12R11]: Thank you, it has been modified as required.

Methods

Study population

During a retrospective cross-sectional analysis, data from 6,588 participants in the NHANES of the United States were examined during the period 1999–2004[14–16]. The NHANES compiles diverse health-related data, including demographic details, physical examination outcomes, laboratory results, and dietary patterns. The National Center for Health Statistics collected this information with the approval of its ethics review board. Before participating in the NHANES, all participants provided written informed consent[17,18]. NHANES data can be accessed by the public through the NHANES website (http://www. cdc. gov/nchs/nhanes. htm). Of the 31,126 individuals who participated in the in-home interview, we excluded those aged below 40 years (n=21,156) because an ankle brachial index (ABI) was not measured in this age group. Participants were excluded from the study if they had missing bilateral ABI data (n=2408),ABI values exceeding 1.4 (n=65) or were lacking diet data (n=169). Individuals with missing covariates were excluded (n=740), resulting in a final analysis sample of 6,588 participants.

Commented [A13]: You should explain the meaning of this abbreviation before use it in the paragraph.

Commented [A14R13]: Thank you, it has been modified as required.

Commented [A15]: The comma is redundant.

Commented [A16R15]: Thank you, it has been modified as required.

Ankle-brachial index

Systolic blood pressure measurements were taken from the right arm (brachial artery) and both ankles (posterior tibial artery) after a brief period of rest. If the participant's right arm measurements were unavailable due to any conditions that could affect accuracy, such as open wounds or dialysis shunts, the left arm was used for the brachial pressure measurement. Individuals aged 40-59 had their systolic blood pressure measured twice, while those aged 60 and older had it measured only once. The ankle-brachial index (ABI) was calculated by dividing the average systolic blood pressure (ASBP) of the ankle by the ASBP in the arm. For participants aged 60 and older, the single reading represented the mean values. ABI <0.9 was used to define PAD[19,20].

Dietary vitamin E intake

Data related to vitamin E intake in diets was gathered from the NHANES 1999 to 2004 first-day dietary interview examination files. A 24-hour dietary recall interview was conducted via the NHANES computer-assisted dietary data interview system to acquire detailed dietary intake data for all participants. This interview captured details of all foods and beverages consumed within a 24-hour duration, such as consumption time, eating occasion, food descriptions, portion sizes, food sources, and location of consumption. Following the dietary recall, a set of health-related inquiries were made. The data collection approach utilized the Automated Multiple Pass Method, which is the dietary data collection tool of the US Department of Agriculture (USDA).Detailed methodologies for the dietary survey can be found in the NHANES Dietary Interviewer's Procedure Manual[15]. While 24-h dietary recalls possess inherent limitations in terms of reliability and effectiveness, they offer a greater level of specificity regarding the varieties and amounts of ingested foods than food frequency questionnaires[21–23]. The participants were then divided into tertile (Q1–Q3) based on their dietary vitamin E consumption.

Commented [A17]: Quintile or tertile?
Why do you prefer the data spilt in quintiles? Please explain.

Commented [A18R17]: Thank you for pointing out the error, in fact the study population was divided into tertile.

Covariates

Standardized questionnaires were administered to obtain socio-demographic and lifestyle information. The Mobile Examination Center furnished the results of the examinations, including

body mass index (BMI), blood pressure, and various biochemical parameters. Different potential covariates were evaluated using the available literature[24], including age, sex, race/ethnicity, education level, marital status, family income, smoking status, physical activity, BMI, laboratory parameters (total cholesterol and glycated hemoglobin A1c), and comorbidities (e.g., diabetes, hypertension, and cardiovascular disease [CVD]). For detailed information regarding these variables, such as the methods used for measurement, questionnaire data, and the list of variables, please refer to the official NHANES website (www.cdc.gov/nchs/nhanes/ accessed on 1 May 2022). The categorization of family income was based on the poverty income ratio (PIR) into three groups: low (PIR ≤ 1.3), medium (PIR > 1.3–3.5), and high (PIR > 3.5). Smoking status was classified according to established definitions from previous studies: never smoked, current smoker, and former smoker. To evaluate physical activity levels, participants were asked about intense exercises leading to notable changes in breathing and heart rate (such as swimming or high-speed cycling), as well as moderate exercises causing mild-to-moderate increases in these physiological parameters (for example, golfing or leisurely biking). The duration of each activity should have been at least 10 min within the last month. Physical activity levels were categorized into three groups: below-moderate (lacking both moderate and intense activities), moderate (absence of intense exercise but inclusion of at least one moderately active pursuit), and high-intensity (presence of at least one episode involving rigorous exertion).

The identification of previous illnesses (such as hypertension and diabetes) was established through a questionnaire by asking participants if they had previously informed a doctor about their medical conditions. The history of cardiovascular disease was determined based on individuals' self-reported experiences with congestive heart failure, coronary heart disease, angina pectoris, heart attack, and stroke. BMI was calculated using a conventional method that considers weight and height.

Statistical analysis

This study used a rigorous and comprehensive approach to assess the effects of dietary vitamin E intake on PAD. Analysis of the association between dietary vitamin E intake and PAD was conducted using robust statistical techniques, such as binary logistic regression models that provide odds ratios and 95% confidence intervals, consideration of confounders based on expert judgment, existing scientific literature, and identification of significant covariates in univariate analysis, indicating a thoughtful approach to controlling potential sources of bias. Additionally, the use of a subgroup analysis to examine the relationship between vitamin E intake and PAD within specific subgroups adds to these findings. The handling of missing data through listwise deletion, sensitivity analyses to assess the robustness of the findings, and the reporting and comparison of effect sizes and p-values from various association inference models further enhanced the credibility and reliability of the study's conclusions. Overall, the methodology and analytical approach contributed to the robustness and validity of the findings.

Statistical analyses were performed using R Statistical Software (Version 4.2.2, http://www.R-project.org, The R Foundation) and the Free Statistics Analysis Platform (Version 1.9, Beijing, China, http://www.clinicalscientists.cn/freestatistics). A detailed explanation of their functionalities ensured transparency and reproducibility of the analytical methods used in this study. Overall, the statistical analysis appears to be comprehensive, well executed, and supported by appropriate software tools, contributing to the strength of the study's findings.

Results

Characteristics of the study population

In total, 31,126 individuals participated in the in-home interviews. Among them, 21,156 participants aged < 40 years were excluded. Furthermore, participants who had missing bilateral ABI data (n=2408), ABI values exceeding 1.4 (n=65), or lacking diet information (n=169) were excluded. Participants with incomplete covariate data were excluded (n=740), resulting in a final sample size of 6,588 participants (Fig 1).

Fig 1. Research flowchart

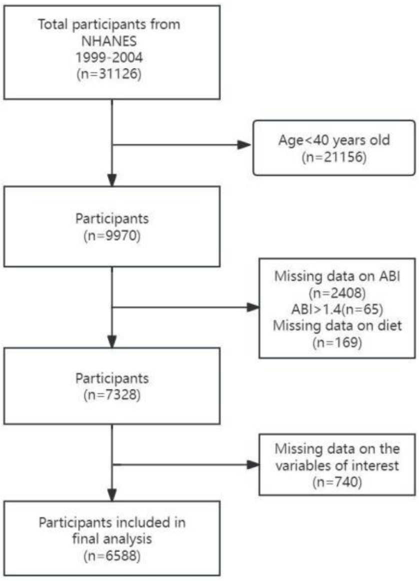

The study included qualified individuals aged 40 years or older with a PAD prevalence rate of 5.9%. Table 1 presents the basic features of the participants categorized according to their dietary consumption of vitamin E. The three groups differed in terms of sex, age, education level, race, MS, PIR, physical activity, HbA1c, smoking status, hypertension, CVD, and diabetes mellitus ($P < 0.05$). However, the distribution of patient characteristics, such as BMI and total cholesterol, among the dietary vitamin E intake groups was similar ($P > 0.05$)(Table 1).

Table 1. Baseline characteristics stratified by the dietary vitamin E intake quartiles (Q).

**Variables**	**Total (n = 6588)**	**Q1 (n = 2191)**	**Q2 (n = 2195)**	**Q3 (n = 2202)**	***p*- Value**
**Gender, n (%)**					< 0.001
Male	3362 (51.0)	951 (43.4)	1086 (49.5)	1325 (60.2)	
Female	3226 (49.0)	1240 (56.6)	1109 (50.5)	877 (39.8)	
**Age (years)**	60.1 ± 13.0	61.6 ± 12.9	60.2 ± 13.2	58.4 ± 12.6	< 0.001
Race, n (%)					< 0.001

Commented [A19]: The form has been revised with reference to the guidelines.

Commented [A20R19]: Thank you, it has been modified as required.

Commented [A21]: Why is this different font?

Commented [A22R21]: Thank you, it has been modified as required.

Commented [A23R21]: Thank you, it has been modified as required.

Mexican American	1371 (20.8)	507 (23.1)	449 (20.5)	415 (18.8)	
Other Hispanic	261 (4.0)	93 (4.2)	84 (3.8)	84 (3.8)	
Non-Hispanic white	3635 (55.2)	1070 (48.8)	1238 (56.4)	1327 (60.3)	
Non-Hispanic black	1127 (17.1)	453 (20.7)	355 (16.2)	319 (14.5)	
Other races	194 (2.9)	68 (3.1)	69 (3.1)	57 (2.6)	
**Education level,, n (%)**					< 0.001
Less than high school	1140 (17.3)	513 (23.4)	352 (16)	275 (12.5)	
High school diploma or GED	2562 (38.9)	908 (41.4)	854 (38.9)	800 (36.3)	
More than high school	2886 (43.8)	770 (35.1)	989 (45.1)	1127 (51.2)	
**MS, n (%)**					< 0.001
Married/Living with partner	4381 (66.5)	1316 (60.1)	1476 (67.2)	1589 (72.2)	
Widowed/Divorced/Separated	1839 (27.9)	746 (34)	604 (27.5)	489 (22.2)	
Never married	368 (5.6)	129 (5.9)	115 (5.2)	124 (5.6)	
**PIR, n (%)**					< 0.001
Low income	1504 (22.8)	635 (29)	490 (22.3)	379 (17.2)	
Medium income	2290 (34.8)	785 (35.8)	764 (34.8)	741 (33.7)	
High income	2794 (42.4)	771 (35.2)	941 (42.9)	1082 (49.1)	
**Physical activity, n (%)**					< 0.001
Sedentary	3017 (45.8)	1190 (54.3)	989 (45.1)	838 (38.1)	
Moderate	2071 (31.4)	629 (28.7)	706 (32.2)	736 (33.4)	
Vigorous	1500 (22.8)	372 (17)	500 (22.8)	628 (28.5)	
**BMI, kg/m2**	28.5 ± 5.6	28.3 ± 5.5	28.4 ± 5.6	28.7 ± 5.6	0.135
**Total cholesterol, mg/dL**	209.6 ± 41.3	210.9 ± 41.9	209.0 ± 41.0	208.7 ± 41.0	0.150
**HbA1c, %**	5.8 ± 1.1	5.8 ± 1.2	5.8 ± 1.0	5.7 ± 1.1	0.003
**Diabetes, n (%)**					
No	5742 (87.2)	1864 (85.1)	1921 (87.5)	1957 (88.9)	
Yes	846 (12,8)	327 (14.9)	274 (12,5)	245 (11.1)	
**Hypertension, n (%)**					0.012
No	4251 (64.5)	1361 (62.1)	1432 (65.2)	1458 (66.2)	
Yes	2337 (35.5)	830 (37.9)	763 (34.8)	744 (33.8)	
**CVD, n (%)**					0.025
No	5546 (84.2)	1809 (82.6)	1854 (84.5)	1883 (85.5)	
Yes	1042 (15.8)	382 (17.4)	341 (15.5)	319 (14.5)	
**Smoking, n (%)**					< 0.001
Never	3043 (46.2)	1011 (46.1)	1038 (47.3)	994 (45.1)	
Former	2288 (34.7)	707 (32.3)	753 (34.3)	828 (37.6)	
Current	1257 (19.1)	473 (21.6)	404 (18.4)	380 (17.3)	
**PAD, n (%)**					< 0.001
No	6199 (94.1)	2023 (92.3)	2054 (93.6)	2122 (96.4)	
Yes	389 (5.9)	168 (7.7)	141 (6.4)	80 (3.6)	

Values for BMI and age Values are given as mean ± standard deviation , other values are presented as numbers and percentages. BMI, body mass index; CVD, cardiovascular disease; CRP, C-reactive protein; HbA1c, glycosylated hemoglobin; MS, marital status; PIR, poverty income ratio; PAD, peripheral arterial disease.

Commented [A24]: Thank you, it has been modified as required.

Association between dietary vitamin E intake and PAD

In the multivariable logistic regression analyses, it was found that participants in the Q3 group exhibited a lower probability of PAD (OR=0.68, 95% CI=0.51–0.91, P=0.009) than the Q1 group, after adjusting for potential confounders (Table 2, model 5).

The adjusted smoothed plots suggested a straightforward linear relationship between dietary vitamin E intake and PAD (Figure 2; P for nonlinearity = 0.897; the highest and lowest 0.5% were trimmed for each dietary vitamin E intake measure). As the dietary vitamin E intake increased, the risk of PAD decreased.

Figure 2 shows the correlation between dietary vitamin E intake and PAD in both males and females. Data were analyzed using a multivariate logistic regression model with restricted cubic splines. To account for various factors, such as sex, age, BMI, severity of illness, and reason for admission (Model 5), dietary vitamin E intake was considered a continuous variable. The reference standard was the median dietary vitamin E intake. Shaded areas represent 95% confidence intervals. The curves highlight the inverse relationship between dietary vitamin E intake and PAD when considering low or near-median values of dietary vitamin E intake. Owing to limited patient numbers and the nature of the cubic fit analysis, there was significant variability in the 95% confidence intervals at extreme values.

Table 2. Odds ratios (ORs) and 95% confidence interval (CI) of the dietary vitaminE intake quartiles for PAD.

Variable	VitaminE(mg) OR (95%CI)	P-value	Q1 (0-4.4) OR (95%CI)	Q2 (4.41-7.69) OR (95%CI)	P-value	Q3 (7.7-97.0) OR (95%CI)	P-value	Trend.test	P-value
**Model 1**	0.944(0.92~0.969)	<0.001	1 (Ref)	0.83 (0.66~1.04)	0.108	0.45 (0.35~0.6)	<0.001	0.69 (0.61~0.79)	<0.001
**Model 2**	0.973 (0.949~0.997)	0.0279	1 (Ref)	0.97 (0.76~1.23)	0.779	0.63 (0.47~0.84)	0.002	0.81 (0.71~0.93)	0.003
**Model 3**	0.975 (0.951~0.999)	0.0406	1 (Ref)	0.97 (0.76~1.24)	0.808	0.64 (0.48~0.86)	0.003	0.82 (0.71~0.94)	0.005
**Model 4**	0.976 (0.952~1)	0.0517	1 (Ref)	0.97 (0.76~1.24)	0.804	0.65 (0.48~0.86)	0.003	0.82 (0.71~0.94)	0.005
**Model 5**	0.981 (0.957~1.004)	0.1075	1 (Ref)	1 (0.78~1.28)	0.992	0.68 (0.51~0.91)	0.009	0.84 (0.73~0.97)	0.016

Data are presented as odds ratios, 95% CIs (confidence intervals), and p-value.
Model 1 adjusted for none.
Model 2 adjusted for age,gender,race, education level,MS, physical activity.
Model 3 adjusted for age,gender,race, education level,MS, physical activity,PIR,BMI,total cholesterol, HbA1c.
Model 4 adjusted for age,gender,race, education level,MS, physical activity,PIR,BMI,total cholesterol,HbA1c, diabetes,hypertension,CVD.
Model 5 adjusted for all covariates.

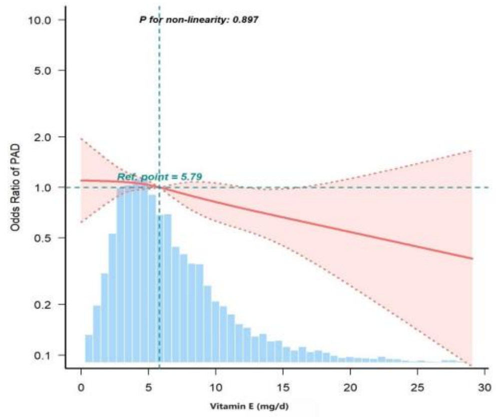

Fig2.Association between dietary vitamin E intake and PAD odds ratio. Solid and dashed lines represent the predicted value and 95% confidence intervals. They were adjusted for age, sex, race/ethnicity, education level, marital status, family income, smoking status, physical activity, BMI, total cholesterol, glycated hemoglobin A1c, and comorbidities. Only 99% of the date is shown.

Stratification analysis

In this study, we performed a subgroup analysis based on various factors, including age, sex, ethnicity, presence of hypertension or diabetes, educational background, history of CVD, income level (PIR), physical activity level, and smoking status. Additionally, sensitivity and supplementary analyses were conducted, and the results of the subgroup analysis indicated no significant evidence of effect modification or interaction based on common risk factors for PAD (all P values for interaction were >0.05) (Fig 3).

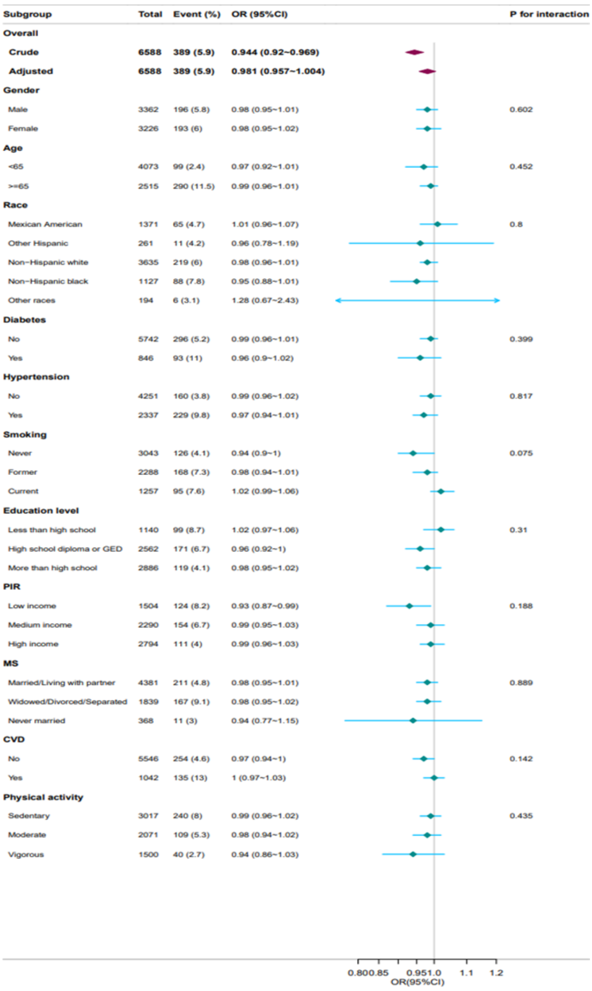

Fig3.The relationship between dietary vitamin E intake and PAD according to basic features. Except for the stratification component itself, each stratification factor was adjusted for all other variables.

Discussion

To the extent of our understanding, in a retrospective cross-sectional study conducted on a significant number of participants involved in the NHANES between 1999 and 2004 in the United States, we consistently observed an inverse correlation between the intake of dietary vitamin E and likelihood of developing PAD. Results were consistent across clinical subgroups and in sensitivity analyses. These observations would have important implications for current PAD management strategies.

Vitamin E, a membrane-bound antioxidant found in nuts, vegetable oils, and fish, is associated with reduced cellular damage in cardiovascular disease[21]. Its antioxidant and anti-inflammatory properties, along with its ability to prevent platelet aggregation and smooth cell proliferation, have been shown to exhibit effects that counteract the development of atherosclerosis [25].

Our research findings align with previous observational studies indicating an inverse relationship between the dietary intake of vitamin E and the occurrence of PAD among participants in the NHANES conducted from 1999 to 2004[9,14,16]. According to a study conducted by Lane et al.[21], an increased intake of vitamin E exhibited a notable safeguarding effect, regardless of the presence of conventional cardiovascular risk factors, within the population of the United States. Not come singly but in pairs, Antonelli-Incalzi et al.[26] conducted a study on the nutrient consumption of 1251 individuals living in their own homes, who were part of the InCHIANTI research. The average age of participants were 68 years. They also investigated whether there was a potential decrease in the risk of PAD associated with a daily intake of Vitamin E equal to or greater than 7.726 mg/d. (OR: 0.37; 95% CI 0.16–0.84). In addition, the Rotterdam Study found that there was a negative correlation between vitamin E consumption and PAD in men. However, it remains unclear whether the association between antioxidant intake and PAD differs between sexes or whether these findings are influenced by the different dietary patterns observed in men than in women [27]. Further research is necessary to validate our results and investigate the intricate relationships and potential mechanisms involved.

Our findings support and expand upon previous research conducted on participants in the NHANES from 1999 to 2004 in the United States. We found that a higher intake of dietary vitamin E was associated with a reduced risk of PAD after accounting for all relevant factors (OR, 0.981; 95% CI, 0.957–1.004). When we divided dietary vitamin E intake into quartiles, individuals in the third quartile had a lower incidence rate of PAD than those in the first quartile (OR, 0.68; 95% CI: 0.51, 0.91). Subgroup analysis yielded similar results without any significant differences (all P values for interactions were >0.05). Extensive clinical research has demonstrated the potential protective effects of vitamin E against long-term cardiovascular diseases[28]. Studies have indicated that taking vitamin E supplements for more than four years can result in a substantial 59% decrease in mortality from coronary disease[29]. The discussion regarding the impact of vitamin E on cardiovascular diseases is complex. While certain studies indicate that vitamin E supplements may provide protection against cardiovascular diseases, others do not support this claim. Additionally, a minority of studies have even suggested potential adverse effects[30,31]. So further investigations are necessary to validate our findings and explore the intricate relationships and potential mechanisms involved.

This was a well-designed retrospective cross-sectional study with a robust sample size and stringent inclusion and exclusion criteria. Baseline data on a wide range of lifestyle factors, including dietary cofactors, were collected, enabling adjustment for potential confounders in the analyses. Additionally, a comprehensive set of sensitivity analyses was conducted to enhance the interpretation of the results and validate the main findings.

However, this study had several limitations. First, owing to the cross-sectional and observational nature of the analysis, causal relationships among dietary vitamin E intake, covariates, and PAD could not be definitively established. Despite efforts to control for relevant confounders in the multivariate model, the presence of unmeasured or unknown residual confounders, such as dietary habits and family income, may have led to an overestimation of the observed associations. Furthermore, in this study, the intake of vitamin E was not energy-adjusted.Nonetheless, the study mainly included American adults, which limits the generalizability of the findings and necessitates further research on diverse populations.

Conclusions

Our study provides supporting evidence indicating a detrimental correlation between the consumption of dietary vitamin E and the occurrence of PAD in US adults older than 40 years. Therefore, individuals with insufficient dietary vitamin E intake, especially those with a very low vitamin E intake, should consider increasing their vitamin E intake to lower the risk of developing PAD. These findings should be considered when offering dietary guidance to prevent PAD .

Commented [A25]: Please specify the age group to which your results should be adapted

Commented [A26R25]: Thank you, it has been modified as required.

Statements

Acknowledgement

We express our gratitude to Jie Liu from the Department of Vascular and Endovascular Surgery at Chinese PLA General Hospital for providing valuable assistance in statistical analysis, study design consultations, and offering insightful feedback on the manuscript.

Statement of Ethics

Study approval statement: The study did not require any additional institutional review board approval for the secondary analysis, thus ethical review and approval were exempted. Consent to participate statement: The NHANES study was approved by the Ethics Review Committee of the National Center for Health Statistics (NCHS), and prior to their involvement, all participants provided written consent after being fully informed.

Conflict of Interest Statement

The authors have no conflicts of interest to declare.

Funding Sources

This study was not financially supported by any funding agencies in the public, commercial, or non-profit sectors.

Author Contributions

Conceptualization, Qiang Liu and Jianjun Shi; Data curation, Qiang Liu and Xing Wu; Formal analysis and Methodology, Qiang Liu, Xing Wu and Jianjun Shi; Funding acquisition, Yun Wang and Jianjun Shi; Writing—original draft, Qiang Liu and Xing Wu; Writing—Xiang Wang and Fei Zhao; All the authors have carefully reviewed and given their consent to the final version of the manuscript.

Data Availability Statement

Publicly accessible datasets for this study can be found online. The name of the repository/repositories and their corresponding accession numbers are provided at http://www.cdc.gov/nchs/nhanes/ (accessed on 1 May 2022).

References

1. Poledniczek M, Neumayer C, Kopp CW, Schlager O, Gremmel T, Jozkowicz A, et al. Micro- and Macrovascular Effects of Inflammation in Peripheral Artery Disease—Pathophysiology and Translational Therapeutic Approaches. Biomedicines. 2023;11:2284.

2. Zhang Z, Chen Z. Higher Systemic Immune-Inflammation Index is Associated With Higher Likelihood of Peripheral Arterial Disease. Ann Vasc Surg. 2022;84:322–6.

3. Hirsch AT, Haskal ZJ, Hertzer NR, Bakal CW, Creager MA, Halperin JL, et al. ACC/AHA 2005 Guidelines for the Management of Patients With Peripheral Arterial Disease (Lower Extremity, Renal, Mesenteric, and Abdominal Aortic): Executive Summary A Collaborative Report From the American Association for Vascular Surgery/Society for Vascular Surgery,∗∗AAVS/SVS when Guideline initiated, now merged into SVS Society for Cardiovascular Angiography and Interventions, Society for Vascular Medicine and Biology, Society of Interventional Radiology, and the ACC/AHA Task Force on Practice Guidelines (Writing Committee to Develop Guidelines for the Management of Patients With Peripheral Arterial Disease). Journal of the American College of Cardiology. 2006;47:1239–312.

4. Correction to: 2024 Heart Disease and Stroke Statistics: A Report of US and Global Data From the American Heart Association. Circulation. 2024;149:e1164.

5. Zhu K, Qian F, Lu Q, Li R, Qiu Z, Li L, et al. Modifiable Lifestyle Factors, Genetic Risk, and Incident Peripheral Artery Disease Among Individuals With Type 2 Diabetes: A Prospective Study. Diabetes Care. 2024;47:435–43.

6. Pan D, Guo J, Su Z, Meng W, Wang J, Guo J, et al. Association of prognostic nutritional index with peripheral artery disease in US adults: a cross-sectional study. BMC Cardiovasc Disord. 2024;24:133.

7. Joosten MM, Pai JK, Bertoia ML, Rimm EB, Spiegelman D, Mittleman MA, et al. Associations between conventional cardiovascular risk factors and risk of peripheral artery disease in men. JAMA. 2012;308:1660–7.

8. Navab M, Ananthramaiah GM, Reddy ST, Van Lenten BJ, Ansell BJ, Fonarow GC, et al. The oxidation hypothesis of atherogenesis: the role of oxidized phospholipids and HDL. J Lipid Res. 2004;45:993–1007.

9. Naqvi AZ, Davis RB, Mukamal KJ. Nutrient intake and peripheral artery disease in adults: key considerations in cross-sectional studies. Clin Nutr. 2014;33:443–7.

10. Min J-Y, Cho J-S, Lee K-J, Park J-B, Park S-G, Kim JY, et al. Potential role for organochlorine pesticides in the prevalence of peripheral arterial diseases in obese persons: results from the National Health and Nutrition Examination Survey 1999-2004. Atherosclerosis. 2011;218:200–6.

11. Selvin E, Köttgen A, Coresh J. Kidney function estimated from serum creatinine and cystatin C and peripheral arterial disease in NHANES 1999-2002. Eur Heart J. 2009;30:1918–25.

12. Colombo ML. An update on vitamin E, tocopherol and tocotrienol-perspectives. Molecules. 2010;15:2103–13.

13. Gahche J, Bailey R, Burt V, Hughes J, Yetley E, Dwyer J, et al. Dietary supplement use among U.S. adults has increased since NHANES III (1988-1994). NCHS Data Brief. 2011;1–8.

14. Wu Z, Ruan Z, Liang G, Wang X, Wu J, Wang B. Association between dietary magnesium intake and peripheral arterial disease: Results from NHANES 1999-2004. PLoS One. 2023;18:e0289973.

15. Hicks CW, Wang D, Matsushita K, McEvoy JW, Christenson R, Selvin E. Glycated albumin and HbA1c as markers of lower extremity disease inUS adults with and without diabetes. Diabetes Res Clin Pract. 2022;184:109212.
16. Qu C-J, Teng L-Q, Liu X-N, Zhang Y-B, Fang J, Shen C-Y. Dose-Response Relationship Between Physical Activity and the Incidence of Peripheral Artery Disease in General Population: Insights From the National Health and Nutrition Examination Survey 1999-2004. Front Cardiovasc Med. 2021;8:730508.
17. Fan H, Zhou J, Huang Y, Feng X, Dang P, Li G, et al. A Proinflammatory Diet Is Associated with Higher Risk of Peripheral Artery Disease. Nutrients. 2022;14.
18. Liu Y, Chang L, Wu M, Xu B, Kang L. Triglyceride Glucose Index Was Associated With the Risk of Peripheral Artery Disease. Angiology. 2022;73:655–9.
19. Loreaux F, Jéhannin P, Le Pabic E, Paillard F, Le Faucheur A, Mahe G. An unfavorable dietary pattern is associated with symptomatic peripheral artery disease. Nutr Metab Cardiovasc Dis. 2024;34:2173–81.
20. Liu Y, Wei R, Tan Z, Chen G, Xu T, Liu Z, et al. Association between dietary fiber intake and peripheral artery disease in hypertensive patients. J Health Popul Nutr. 2024;43:118.
21. Lane JS, Magno CP, Lane KT, Chan T, Hoyt DB, Greenfield S. Nutrition impacts the prevalence of peripheral arterial disease in the United States. Journal of Vascular Surgery. 2008;48:897-904.e1.
22. Mazidi M, Mikhailidis DP, Banach M. Higher dietary acid load is associated with higher likelihood of peripheral arterial disease among American adults. J Diabetes Complications. 2018;32:565–9.
23. Mattioli AV, Francesca C, Mario M, Alberto F. Fruit and vegetables in hypertensive women with asymptomatic peripheral arterial disease. Clin Nutr ESPEN. 2018;27:110–2.
24. Cheng Y, Fang Z, Zhang X, Wen Y, Lu J, He S, et al. Association between triglyceride glucose-body mass index and cardiovascular outcomes in patients undergoing percutaneous coronary intervention: a retrospective study. Cardiovasc Diabetol. 2023;22:75.
25. Kumar M, Deshmukh P, Kumar M, Bhatt A, Sinha AH, Chawla P. Vitamin E Supplementation and Cardiovascular Health: A Comprehensive Review. Cureus [Internet]. 2023 [cited 2024 Mar 10]; Available from: https://www.cureus.com/articles/184881-vitamin-e-supplementation-and-cardiovascular-health-a-comprehensive-review.
26. Antonelli-Incalzi R, Pedone C, McDermott MM, Bandinelli S, Miniati B, Lova RM, et al. Association between nutrient intake and peripheral artery disease: Results from the InCHIANTI study. Atherosclerosis. 2006;186:200–6.
27. Klipstein-Grobusch K. Dietary Antioxidants and Peripheral Arterial Disease : The Rotterdam Study. American Journal of Epidemiology. 2001;154:145–9.
28. Azzi A, Meydani SN, Meydani M, Zingg JM: The rise, the fall and the renaissance of vitamin E . Arch Biochem Biophys. 2016, 595:100-8.
29. Muntwyler J, Hennekens CH, Manson JE, Buring JE, Gaziano JM: Vitamin supplement use in a low-risk population of US male physicians and subsequent cardiovascular mortality . Arch Intern Med. 2002, 162:1472-6.
30. Mezzetti A, Zuliani G, Romano F, Costantini F, Pierdomenico SD, Cuccurullo F, Fellin R:

Vitamin E and lipid peroxide plasma levels predict the risk of cardiovascular events in a group of healthy very old people . J Am Geriatr Soc. 2001, 49:533-7.

31. Wright ME, Lawson KA, Weinstein SJ, Pietinen P, Taylor PR, Virtamo J, Albanes D: Higher baseline serum concentrations of vitamin E are associated with lower total and cause-specific mortality in the AlphaTocopherol, Beta-Carotene Cancer Prevention Study . Am J Clin Nutr. 2006, 84:1200-7.

Dear Editor and Reviewers:
On behalf of my co-authors, we greatly appreciate the careful review and comments from both you and the reviewers. We believe that by implementing the suggested changes, we now have a stronger manuscript entitled “Association of dietary vitamin E intake with peripheral arterial disease: A retrospective cross-sectional study” for submission to Journal of PLOS ONE. We look forward to your positive response to the revised work submitted here.
We present here point-to-point responses for each of the comments in the attached document and have revised our manuscript accordingly. We do not change our statistics or results. And we hope the revised manuscript could be acceptable to you. Revised sections are identified with red text in the paper.
There are no conflicts of interest regarding this work. All authors have read the revised manuscript and approved its submission to the journal “PLOS ONE”. Please do not hesitate to contact us if you have any questions.
Thank you and best regards.
Yours Sincerely,
Jianjun Shi
mail: 13934282361@163.com
Department of Cardiovascular Surgery,Taiyuan Central Hospital; Department of Cardiovascular Surgery, the Ninth Clinical College Affiliated to Shanxi Medical University, Taiyuan,030000, China.

Responds to the reviewer’s comments:

Reviewer #1:
Dear Reviewer,
Thank you very much for your time involved in reviewing the manuscript and your comments have greatly helped to improve the quality of our manuscript.

Comments: This is a population study whose methodology was presented in a coherent and well-founded manner, thereby providing a robust foundation for the authors' findings.The following considerations pertain to adjustments:
1. The acronym "ABI" should be written in full for the first time it is mentioned in the text.
2. While the methods of the primary study are duly referenced, it is nevertheless necessary to provide a detailed account of the methodology employed for the assessment of vitamin E consumption in this manuscript. Please indicate the number of 24-hour dietary recalls utilized in the study. Please clarify whether vitamin E intake was corrected for energy. It would be beneficial to ascertain whether the participants were taking a vitamin E supplement.
3. As noted, Reference 14 examined the same population and investigated the correlation between vitamin E intake and DAP. In what ways does your study differ from that of Reference 14?
4. The majority of the references utilized in this study are old, with 80% of them published

more than five years ago. It is recommended that more current references be cited, particularly those used in the discussion to support the relationship between vitamin E consumption and DAP.

5. In line 228, the text makes reference to "recent studies," yet no corresponding reference is provided. Please provide the references for these recent studies.

6. The observational, cross-sectional study does not define a causal relationship. It is therefore advisable to exercise caution when considering the phrase "These findings should be used when formulating clinical guidelines."

7. The extant literature indicates that the isolated consumption of vitamin E does not provide cardiovascular protection. This is evidenced by the fact that studies that used supplementation did not observe the same results as observational studies. It is therefore of interest that this topic is introduced for discussion.

Comment 1:

The acronym "ABI" should be written in full for the first time it is mentioned in the text.

Respond 1:

Thank you for the valuable suggestion. Firstly, as suggested by you, the abbreviation ABI has been written in full and explained before its first use; Secondly, PAD is defined in the last sentence of the paragraph. As follows:

Ankle-brachial index

Systolic blood pressure measurements were taken from the right arm (brachial artery) and both ankles (posterior tibial artery) after a brief period of rest. If the participant's right arm measurements were unavailable due to any conditions that could affect accuracy, such as open wounds or dialysis shunts, the left arm was used for the brachial pressure measurement. Individuals aged 40-59 had their systolic blood pressure measured twice, while those aged 60 and older had it measured only once. The ankle-brachial index (ABI) was calculated by dividing the average systolic blood pressure (ASBP) of the ankle by the ASBP in the arm. For participants aged 60 and older, the single reading represented the mean values. ABI <0.9 was used to define PAD.

Comment 2:

While the methods of the primary study are duly referenced, it is nevertheless necessary to provide a detailed account of the methodology employed for the assessment of vitamin E consumption in this manuscript. Please indicate the number of 24-hour dietary recalls utilized in the study. Please clarify whether vitamin E intake was corrected for energy. It would be beneficial to ascertain whether the participants were taking a vitamin E supplement.

Respond 2:

Thank you for the detailed review. Firstly,based on your this comment, we have added a description of the methodology used to assess vitamin E intake. Furthermore, in this study, the intake of vitamin E was not energy-adjusted, and this limitation has been addressed in the discussion. In addition, data related to vitamin E intake in diets was gathered from the

NHANES 1999 to 2004 first-day dietary interview examination files. This interview captured details of all foods and beverages consumed within a 24-hour duration, such as consumption time, eating occasion, food descriptions, portion sizes, food sources, and location of consumption,and did not contain vitamin E supplement . As follows:

Data related to vitamin E intake in diets was gathered from the NHANES 1999 to 2004 first-day dietary interview examination files. A 24-hour dietary recall interview was conducted via the NHANES computer-assisted dietary data interview system to acquire detailed dietary intake data for all participants. This interview captured details of all foods and beverages consumed within a 24-hour duration, such as consumption time, eating occasion, food descriptions, portion sizes, food sources, and location of consumption. Following the dietary recall, a set of health-related inquiries were made. The data collection approach utilized the Automated Multiple Pass Method, which is the dietary data collection tool of the US Department of Agriculture (USDA).

Comment 3:
As noted, Reference 14 examined the same population and investigated the correlation between vitamin E intake and DAP. In what ways does your study differ from that of Reference 14?
Respond 3:
Thank you for the detailed review. The differences between my study and Reference 14 are as follows:
1. My study provides a research flowchart, demonstrating the design rationale of the study;
2. My study includes curve fitting graphs, making the study results more intuitive;
3. The covariates in my study include age, sex, race, marital status, education, physical activity, income, smoking, hypertension, diabetes, cardiovascular disease, body mass index, total cholesterol, and HbA1c, enhancing the reliability of the study;
4. My study further strengthens the stability of the results through subgroup analysis and forest plots. These are all significant improvements that can enhance the scientific rigor and credibility of the study.

Comment 4:
The majority of the references utilized in this study are old, with 80% of them published more than five years ago. It is recommended that more current references be cited, particularly those used in the discussion to support the relationship between vitamin E consumption and DAP.
Respond 4:
Thank you for the valuable suggestion. Based on your advice, the references in the study have been updated and supplemented, with literature published within the last 5 years accounting for 45.2%. However, the literature on the relationship between vitamin E and PAD published in the past 5 years is limited.
2.Zhang Z, Chen Z. Higher Systemic Immune-Inflammation Index is Associated With Higher Likelihood of Peripheral Arterial Disease. Ann Vasc Surg. 2022;84:322–6.
5.Zhu K, Qian F, Lu Q, Li R, Qiu Z, Li L, et al. Modifiable Lifestyle Factors, Genetic Risk,

and Incident Peripheral Artery Disease Among Individuals With Type 2 Diabetes: A Prospective Study. Diabetes Care. 2024;47:435–43.
6.Pan D, Guo J, Su Z, Meng W, Wang J, Guo J, et al. Association of prognostic nutritional index with peripheral artery disease in US adults: a cross-sectional study. BMC Cardiovasc Disord. 2024;24:133.
15.Hicks CW, Wang D, Matsushita K, McEvoy JW, Christenson R, Selvin E. Glycated albumin and HbA1c as markers of lower extremity disease inUS adults with and without diabetes. Diabetes
Res Clin Pract. 2022;184:109212.
16.Qu C-J, Teng L-Q, Liu X-N, Zhang Y-B, Fang J, Shen C-Y. Dose-Response Relationship Between Physical Activity and the Incidence of Peripheral Artery Disease in General Population:
Insights From the National Health and Nutrition Examination Survey 1999-2004. Front Cardiovasc Med. 2021;8:730508.
18.Liu Y, Chang L, Wu M, Xu B, Kang L. Triglyceride Glucose Index Was Associated With the Risk of Peripheral Artery Disease. Angiology. 2022;73:655–9.
19.Loreaux F, Jéhannin P, Le Pabic E, Paillard F, Le Faucheur A, Mahe G. An unfavorable dietary pattern is associated with symptomatic peripheral artery disease. Nutr Metab Cardiovasc Dis. 2024;34:2173–81.
20.Liu Y, Wei R, Tan Z, Chen G, Xu T, Liu Z, et al. Association between dietary fiber intake and peripheral artery disease in hypertensive patients. J Health Popul Nutr. 2024;43:118.
24.Cheng Y, Fang Z, Zhang X, Wen Y, Lu J, He S, et al. Association between triglyceride glucose-body mass index and cardiovascular outcomes in patients undergoing percutaneous coronary intervention: a retrospective study. Cardiovasc Diabetol. 2023;22:75.
25.Kumar M, Deshmukh P, Kumar M, Bhatt A, Sinha AH, Chawla P. Vitamin E Supplementation and Cardiovascular Health: A Comprehensive Review. Cureus [Internet]. 2023 [cited 2024 Mar 10]; Available from: https://www.cureus.com/articles/184881-vitamin-e-supplementation-and- cardiovascular-health-a-comprehensive-review.

Comment 5:
In line 228, the text makes reference to "recent studies," yet no corresponding reference is provided. Please provide the references for these recent studies.
Respond 5:
Thank you for the valuable suggestion. This sentence is redundant, it has been deleted.

Comment 6:
The observational, cross-sectional study does not define a causal relationship. It is therefore advisable to exercise caution when considering the phrase "These findings should be used when formulating clinical guidelines."
Respond 6:
Thank you for the valuable suggestion. This sentence is indeed not precise, it has been revised as follows: These findings should be considered when offering dietary guidance to prevent PAD.

Comment 7:

The extant literature indicates that the isolated consumption of vitamin E does not provide cardiovascular protection. This is evidenced by the fact that studies that used supplementation did not observe the same results as observational studies. It is therefore of interest that this topic is introduced for discussion.

Respond 7:

Thank you for the detailed review. I have already supplemented as per your request as follows:

Extensive clinical research has demonstrated the potential protective effects of vitamin E against long-term cardiovascular diseases[28]. Studies have indicated that taking vitamin E supplements for more than four years can result in a substantial 59% decrease in mortality from coronary disease[29]. The discussion regarding the impact of vitamin E on cardiovascular diseases is complex. While certain studies indicate that vitamin E supplements may provide protection against cardiovascular diseases, others do not support this claim. Additionally, a minority of studies have even suggested potential adverse effects[30,31]. So further investigations are necessary to validate our findings and explore the intricate relationships and potential mechanisms involved.

Reviewer #2:

Dear Reviewer,

Thank you very much for your time involved in reviewing the manuscript and your comments have greatly helped to improve the quality of our manuscript.

Comments: It is a well-written but incomplete article. Language editing is strongly recommended. Professional language support is recommended. In addition to the grammar check, the statistical as well as grammatical reasons why certain methods are favoured should be elaborated.

Respond：Thank you for the valuable suggestion. I have made the modifications to the grammatically incorrect parts as per your suggestion. Thank you once again for your guidance. After referring to a large number of previous literatures, the statistical methods used in this study include curve fitting, single factor analysis, multiple factor analysis, stratified analysis, subgroup analysis, etc.

1. Through the interpolation algorithm, curve fitting can generate a smooth curve between the given data points, thereby better understanding the trend and pattern of the data；
2. Single factor analysis not only helps in the initial exploration of the relationship between the predictor and response variables, aiding researchers in understanding the impact of individual variables on the outcome. It also assists researchers in selecting the variables that need to be included in the multiple factor analysis, for instance, by evaluating the changes in partial regression coefficients or OR values.
3. Univariate analysis primarily explores the impact of a single independent variable on the dependent variable through t-tests or analysis of variance (ANOVA). The t-test is suitable for comparing the means of two independent samples or paired samples, while ANOVA is used for comparing the means of three or more groups. These two methods

are concise and straightforward, and can intuitively reveal the significant impact of a specific factor when acting independently on the outcome.

4. Multiple factor analysis uses multiple regression analysis to simultaneously examine the combined impact of multiple independent variables on the dependent variable and the interaction effects among the independent variables. This method can more comprehensively reflect the actual situation and reveal the hidden relationships behind complex data. Through multiple regression analysis, we can obtain the regression coefficients of each independent variable, thereby quantifying its specific contribution to the dependent variable, providing strong support for in-depth research.
5. Stratified analysis involves dividing the study subjects into strata based on one or more variables that need to be controlled, which can reduce the impact of confounding factors on the research results and thus provide a more accurate estimation of the association between the exposure factor and the outcome variable.
6. The main purpose of subgroup analysis is to study interactions or effect modification, i.e., whether the effect size varies in different populations or under different conditions. By dividing the study subjects into different subgroups based on certain characteristics (such as gender, disease severity, etc.), estimating the effect sizes for each subgroup separately, and conducting comparisons between subgroups, a better understanding of specific patient, intervention type, or study-specific issues can be achieved.
