# Supplementary material for: Association of dietary vitamin E intake with peripheral arterial disease: A retrospective cross-sectional study
Source: PLoS One. 2025 Mar 24;20(3):e0320356. doi: 10.1371/journal.pone.0320356 (PMC11932459; doi:10.1371/journal.pone.0320356)
Supplement: Attachment — Submitted filename: Response_to_Reviewers_auresp_2.docx [file pone.0320356.s005.docx]

Dear Editor and Reviewers:

On behalf of my co-authors, we greatly appreciate the careful review and comments from both you and the reviewers. We believe that by implementing the suggested changes, we now have a stronger manuscript entitled “Association of dietary vitamin E intake with peripheral arterial disease: A retrospective cross-sectional study” for submission to Journal of PLOS ONE. We look forward to your positive response to the revised work submitted here.

We present here point-to-point responses for each of the comments in the attached document and have revised our manuscript accordingly. We do not change our statistics or results. And we hope the revised manuscript could be acceptable to you. Revised sections are identified with red text in the paper.

There are no conflicts of interest regarding this work. All authors have read the revised manuscript and approved its submission to the journal “PLOS ONE”. Please do not hesitate to contact us if you have any questions.

Thank you and best regards.

Yours Sincerely,

Jianjun Shi

mail: 13934282361@163.com

Department of Cardiovascular Surgery,Taiyuan Central Hospital; Department of Cardiovascular Surgery, the Ninth Clinical College Affiliated to Shanxi Medical University, Taiyuan,030000, China.

Responds to the reviewer’s comments:

**Reviewer #1:** **(No Response)**

**Reviewer #2:**

Dear Author
I've checked your corrections. I still have two suggestions. Firstly, please review the journal's reference citation rules. (Reference 4 is not appropriate.) Secondly, in the conclusion section, not only nutrition guidelines can be emphasised but also nutrition education can be emphasised.

**Respond：**Thank you for the detailed review. Firstly,based on your this comment, we have revised the format of the fourth reference according to the citation rules of the journal.

Secondly, the conclusion section has also been revised according to your suggestions.

1. Martin SS, Aday AW, Almarzooq ZI, Anderson CAM, Arora P, Avery CL, et al. 2024 Heart Disease and Stroke Statistics: A Report of US and Global Data From the American Heart Association. Circulation. 2024;149:e347–913.
2. These findings should be considered when offering dietary guidance and nutrition education to prevent PAD.
