# Supplementary material for: Association of dietary vitamin E intake with peripheral arterial disease: A retrospective cross-sectional study
Source: PLoS One. 2025 Mar 24;20(3):e0320356. doi: 10.1371/journal.pone.0320356 (PMC11932459; doi:10.1371/journal.pone.0320356)
Supplement: Attachment — Submitted filename: PONE-D-24-23667_reviewer NO.docx [file pone.0320356.s001.docx]

PLOS ONE

**Association of dietary vitamin E intake with peripheral arterial disease: A retrospective cross-sectional study**

--Manuscript Draft--

| **Manuscript Number:** | PONE-D-24-23667 |
| --- | --- |
| **Article Type:** | Research Article |
| **Full Title:** | Association of dietary vitamin E intake with peripheral arterial disease: A retrospective cross-sectional study |
| **Short Title:** | Dietary Vitamin E Intake and Peripheral Arterial Disease |
| **Corresponding Author:** | Qiang Liu  Taiyuan City Central Hospital Taiyuan, CHINA |
| **Keywords:** |  |
| **Abstract:** | Introduction: The relationship between the amount of dietary vitamin E consumed and the development of peripheral arterial disease (PAD) remains a topic of debate. This relationship is of the utmost importance in the realms of healthcare and public health and is currently a highly researched and prominent topic.  This study aimed to gain deeper insights into the relationship between dietary vitamin E intake and PAD.  Methods: In a retrospective cross-sectional analysis, data from 6,588 participants in the National Health and Nutrition Examination Survey of the United States were examined during the period 1999–2004. We collected data on age, sex, race, marital status, education, physical activity, income, smoking, hypertension, diabetes, cardiovascular disease, body mass index, total cholesterol and HbA1c. Logistic regression and smooth curve fitting were used to support the research objectives.  Results: After accounting for all relevant factors, a negative correlation between dietary vitamin E intake and the likelihood of PAD was observed (OR: 0.981, 95% CI: 0.957–1.004). The overall prevalence of PAD was 5.9%, with 49.6% in males and 50.4% in females. Individuals in the third quartile of dietary vitamin E intake had a lower occurrence rate of peripheral artery disease than those in the first quartile (OR: 0.68, 95% CI: 0.51, 0.91). Similar patterns of association were observed in the subgroup analysis (all P values for interaction were >0.05).  Conclusions:Our study suggests a negative association between dietary vitamin E intake and incidence of PAD. Therefore individuals with insufficient dietary vitamin E intake, especially those with a very low intake, should consider increasing their vitamin E intake to lower the risk of developing PAD. These findings should be considered while formulating clinical guidelines. |
| **Order of Authors:** | Qiang Liu |
|  | Xing Wu |
|  | Jianjun Shi |
|  | Yun Wang |
|  | Fei Zhao |
|  | Xiang Wang |
| **Additional Information:** |  |
| **Question** | **Response** |
| **Financial Disclosure**  Enter a financial disclosure statement that describes the sources of funding for the work included in this submission. Review the [submission guidelines](http://journals.plos.org/plosone/s/submission-guidelines#loc-financial-disclosure-statement) for detailed | The author(s) received no specific funding for this work. |

*Powered by Editorial Manager® and ProduXion Manager® from Aries Systems Corporation*

| requirements. View published research articles from [PLOS ONE](https://journals.plos.org/plosone/) for specific examples.  This statement is required for submission and **will appear in the published article** if the submission is accepted. Please make sure it is accurate. |  |
| --- | --- |
| **Funded studies**  Enter a statement with the following details:   - Initials of the authors who received each award - Grant numbers awarded to each author - The full name of each funder - URL of each funder website - Did the sponsors or funders play any role in the study design, data collection and analysis, decision to publish, or preparation of the manuscript? |  |
| Did you receive funding for this work? |  |
| **Competing Interests**  Use the instructions below to enter a competing interest statement for this submission. On behalf of all authors, disclose any [competing interests](http://journals.plos.org/plosone/s/competing-interests) that could be perceived to bias this work—acknowledging all financial support and any other relevant financial or non- financial competing interests.  This statement is required for submission and **will appear in the published article** if the submission is accepted. Please make sure it is accurate and that any funding sources listed in your Funding Information later in the submission form are also declared in your Financial Disclosure statement.  View published research articles from  [PLOS ONE](http://journals.plos.org/plosone/) for specific examples. | The authors have declared that no competing interests exist. |

*Powered by Editorial Manager® and ProduXion Manager® from Aries Systems Corporation*

| **NO authors have competing interests**  Enter: The authors have declared that no competing interests exist.  **Authors with competing interests**  Enter competing interest details beginning with this statement:  I have read the journal's policy and the authors of this manuscript have the following competing interests: [insert competing interests here] |  |
| --- | --- |
| * typeset |  |
| **Ethics Statement**  Enter an ethics statement for this submission. This statement is required if the study involved:   - Human participants - Human specimens or tissue - Vertebrate animals or cephalopods - Vertebrate embryos or tissues - Field research   Write "N/A" if the submission does not require an ethics statement.  General guidance is provided below. Consult the [submission guidelines](http://journals.plos.org/plosone/s/submission-guidelines#loc-guidelines-for-specific-study-types) for detailed instructions. **Make sure that all information entered here is included in the Methods section of the manuscript.** | Study approval statement: The study did not require any additional institutional review board approval for the secondary analysis, thus ethical review and approval were exempted.  Consent to participate statement: The NHANES study was approved by the Ethics Review Committee of the National Center for Health Statistics (NCHS), and prior to their involvement, all participants provided written consent after being fully informed. |

*Powered by Editorial Manager® and ProduXion Manager® from Aries Systems Corporation*

| **Format for specific study types**  **Human Subject Research (involving human participants and/or tissue)**   - Give the name of the institutional review board or ethics committee that approved the study - Include the approval number and/or a statement indicating approval of this research - Indicate the form of consent obtained (written/oral) or the reason that consent was not obtained (e.g. the data were analyzed anonymously)   **Animal Research (involving vertebrate animals, embryos or tissues)**   - Provide the name of the Institutional Animal   Care and Use Committee (IACUC) or other relevant ethics board that reviewed the study protocol, and indicate whether they approved this research or granted a formal waiver of ethical approval   - Include an approval number if one was obtained - If the study involved non-human primates, add additional details about animal welfare and steps taken to ameliorate suffering - If anesthesia, euthanasia, or any kind of animal sacrifice is part of the study, include briefly which substances and/or methods were applied   **Field Research**  Include the following details if this study involves the collection of plant, animal, or other materials from a natural setting:   - Field permit number - Name of the institution or relevant body that granted permission |  |
| --- | --- |
|  |  |
| **Data Availability**  Authors are required to make all data underlying the findings described fully available, without restriction, and from the time of publication. PLOS allows rare exceptions to address legal and ethical concerns. See the [PLOS Data Policy](http://journals.plos.org/plosone/s/data-availability) and [FAQ](http://journals.plos.org/plosone/s/data-availability#loc-faqs-for-data-policy) for detailed information. | Yes - all data are fully available without restriction |

*Powered by Editorial Manager® and ProduXion Manager® from Aries Systems Corporation*

| A Data Availability Statement describing where the data can be found is required at submission. Your answers to this question constitute the Data Availability Statement and **will be published in the article**, if accepted. |  |
| --- | --- |
| **Important:** Stating ‘data available on request from the author’ is not sufficient. If your data are only available upon request, select ‘No’ for the first question and explain your exceptional situation in the text box. |  |
| Do the authors confirm that all data underlying the findings described in their manuscript are fully available without restriction? |  |
| **Describe where the data may be found in full sentences. If you are copying our sample text, replace any instances of XXX with the appropriate details.**   - If the data are **held or will be held in a public repository**, include URLs, accession numbers or DOIs. If this information will only be available after acceptance, indicate this by ticking the box below. For example: All XXX files are available from the XXX database (accession number(s) XXX, XXX.). - If the data are all contained **within the**   **manuscript and/or Supporting Information files**, enter the following: All relevant data are within the manuscript and its Supporting Information files.   - If neither of these applies but you are able to provide **details of access elsewhere**, with or without limitations, please do so. For example:   Data cannot be shared publicly because of [XXX]. Data are available from the XXX Institutional Data Access / Ethics Committee (contact via XXX) for researchers who meet the criteria for access to confidential data.  The data underlying the results presented in the study are available from (include the name of the third party | Publicly accessible datasets for this study can be found online. The name of the repository/repositories and their corresponding accession numbers are provided at <http://www.cdc.gov/nchs/nhanes/> (accessed on 1 May 2022). |

*Powered by Editorial Manager® and ProduXion Manager® from Aries Systems Corporation*

| and contact information or URL).   - This text is appropriate if the data are owned by a third party and authors do not have permission to share the data.   * typeset |  |
| --- | --- |
| Additional data availability information: |  |

*Powered by Editorial Manager® and ProduXion Manager® from Aries Systems Corporation*

Manuscript *手稿	[Click here to access/download;Manuscript;VE and PAD](https://www2.cloud.editorialmanager.com/pone/download.aspx?id=36588156&guid=c30b3377-b3f1-4924-be38-dcb9ed14d4e3&scheme=1)[<https://www2.cloud.editorialmanager.com/pone/download.aspx?id=36588156&guid=c30b3377-b3f1-4924-be38-dcb9ed14d4e3&scheme=1> submit.docx](https://www2.cloud.editorialmanager.com/pone/download.aspx?id=36588156&guid=c30b3377-b3f1-4924-be38-dcb9ed14d4e3&scheme=1)

1 ***Research Article***
2 ***Association of dietary vitamin E intake with peripheral arterial disease:***
3 ***A retrospective cross-sectional study***

4

5	Qiang Liu^a,b,1^, Xing Wu^c,d,1^, Yun Wang^a,b^, Xiang Wang^a,b^, Fei Zhao^a,b^, Jianjun Shi^a,b,*^

6

7 ^a^ Department of Cardiovascular Surgery, Taiyuan Central Hospital,China
8 ^b^ Department of Cardiovascular Surgery, the Ninth Clinical College Affiliated to Shanxi Medical
9 University, China
10 ^c^ Department of Nephrology, Taiyuan Central Hospital, China
11 ^d^ Department of Nephrology, the Ninth Clinical College Affiliated to Shanxi Medical University, China

12

13

14	Short Title: to be used as running head: Dietary Vitamin E Intake and Peripheral Arterial Disease

15

16 * Corresponding author. Department of Cardiovascular Surgery, Taiyuan Central Hospital,China;
17 Department of Cardiovascular Surgery, the Ninth Clinical College Affiliated to Shanxi Medical
18 University, China.

19	E-mail address: 13934282361@163.com,,

20

21 Keywords: dietary vitamin E intake; peripheral arterial disease; ankle-brachial index; National Health
22 and Nutrition Examination Survey; cross-sectional study

23

24

25

26

27

28

29

# 30 Abstract

31 Introduction: The relationship between the amount of dietary vitamin E consumed and the
32 development of peripheral arterial disease (PAD) remains a topic of debate. This relationship is of the
33 utmost importance in the realms of healthcare and public health and is currently a highly researched
34 and prominent topic.
35 This study aimed to gain deeper insights into the relationship between dietary vitamin E intake and
36 PAD.
37 Methods: In a retrospective cross-sectional analysis, data from 6,588 participants in the National
38 Health and Nutrition Examination Survey of the United States were examined during the period
39 1999–2004. We collected data on age, sex, race, marital status, education, physical activity, income,
40 smoking, hypertension, diabetes, cardiovascular disease, body mass index, total cholesterol and
41 HbA1c. Logistic regression and smooth curve fitting were used to support the research objectives.
42 Results: After accounting for all relevant factors, a negative correlation between dietary vitamin E
43 intake and the likelihood of PAD was observed (OR: 0.981, 95% CI: 0.957–1.004). The overall
44 prevalence of PAD was 5.9%, with 49.6% in males and 50.4% in females. Individuals in the third
45 quartile of dietary vitamin E intake had a lower occurrence rate of peripheral artery disease than
46 those in the first quartile (OR: 0.68, 95% CI: 0.51, 0.91). Similar patterns of association were observed
47 in the subgroup analysis (all P values for interaction were >0.05).
48 Conclusions: Our study suggests a negative association between dietary vitamin E intake and
49 incidence of PAD. Therefore individuals with insufficient dietary vitamin E intake, especially those
50 with a very low intake, should consider increasing their vitamin E intake to lower the risk of
51 developing PAD. These findings should be considered while formulating clinical guidelines.

52

53

54

55

56

57

58

59

60

## 61 Introduction

62 Peripheral arterial disease (PAD) is caused by atherosclerosis, a condition characterized by the
63 accumulation of fatty deposits in the arteries of the lower extremities and feet ^[1]^ . It is a prevalent
64 disorder affecting ,over 200 million individuals worldwide ^[2]^. In the United States, PAD affects around
65 8.5 million adults aged 40 years and above, with similar occurrence rates among men and women ^[3,4]^.
66 The economic and health burdens of PAD are substantial, emphasizing the need to identify its causes
67 and implement appropriate measures. Previous studies have highlighted the significant roles of
68 inflammatory markers, endothelial dysfunction, and oxidative stress in PAD development^[5,6]^.
69 Although smoking, diabetes, hypertension, and dyslipidemia are established risk factors for PAD^[3,7]^,
70 the influence of diet is unclear. Further investigations are warranted to enhance our understanding of
71 preventive measures and management strategies for PAD, given the limited knowledge of effective
72 preventive factors.
73 Vitamin E, an antioxidant that dissolves in fat, is recognized as a key player in reducing lipid
74 peroxidation in experimental settings^[8]^. Gey et al. first proposed the idea of vitamin E's potential in
75 preventing cardiovascular disease in the "antioxidant hypothesis of atherosclerosis" ^[9]^. Subsequent
76 evidence linked vitamin E to atherosclerosis and thrombotic complications, attributing its anti-
77 atherosclerotic activity to a range of biological functions, including antioxidant functions as
78 scavengers of free radicals, in addition to non-antioxidant roles, such as controlling signal
79 transduction, cellular proliferation, and gene expression. Vitamin E has attracted interest because of
80 its anti-atherogenic and antioxidant properties, which have been associated with improved
81 cardiovascular health in numerous studies. There was a significant increase in alpha-tocopherol levels
82 ^[14–16]^.However, while some studies indicate that supplemental vitamin E may offer protection against
83 cardiovascular diseases, others do not, and a few even suggest the potential for adverse effects^[10,11]^.
84 Therefore, to address this knowledge gap, the ultimate goal was to gain a deeper understanding of
85 how dietary vitamin E intake influences PAD and the implications of this relationship. We sought to
86 gain deeper insights into the relationship between dietary vitamin E intake and PAD through a
87 retrospective cross-sectional study involving 6,588 participants in the National Health and Nutrition
88 Examination Survey (NHANES). We aimed to provide valuable information for personalized nutritional
89 management strategies and contribute to a deeper understanding of the nutritional status of
90 individuals and populations.

## 91 Methods

92

93 Study population
94 During a retrospective cross-sectional analysis, data from 6,588 participants in the NHANES of the
95 United States were examined during the period 1999–2004^[12]^. The NHANES compiles diverse health-
96 related data, including demographic details, physical examination outcomes, laboratory results, and
97 dietary patterns. The National Center for Health Statistics collected this information with the approval
98 of its ethics review board. Before participating in the NHANES, all participants provided written
99 informed consent^[13]^. NHANES data can be accessed by the public through the NHANES website
100 ([http://www.](http://www/) cdc. gov/nchs/nhanes. htm). Of the 31,126 individuals who participated in the in-home
101 interview, we excluded those aged below 40 years (n=21,156) because ABI was not measured in this
102 age group. Participants were excluded from the study if they had missing bilateral ABI data (n=2408),
103 ABI values exceeding 1.4 (n=65), or were lacking diet data (n=169). Individuals with missing covariates
104 were excluded (n=740), resulting in a final analysis sample of 6,588 participants.
105 PAD
106 ABI measurements were conducted in participants aged over 40 years using a specific method
107 outlined in previous studies. Systolic blood pressure readings were obtained from the right arm and
108 both ankles after a brief rest period. When data for the right arm were unavailable, measurements
109 were obtained from the left arm. For individuals aged between 40 and 59 years, systolic blood
110 pressure was assessed on two occasions, whereas for those aged 60 years and above, it was
111 evaluated once. The average systolic blood pressure of the ankle was divided by the arm to calculate
112 the ABI. An ABI <0.9 was used to define PAD^[14–16]^.
113 Dietary vitamin E intake
114 The NHANES dietary survey assessed the types and quantities of foods and drinks consumed by
115 participants within a 24-h period. These data were used to calculate precise nutrient values based on
116 the participants' dietary intake. Detailed methodologies for the dietary survey can be found in the
117 NHANES Dietary Interviewer’s Procedure Manual ^[17]^. While 24-h dietary recalls possess inherent
118 limitations in terms of reliability and effectiveness, they offer a greater level of specificity regarding
119 the varieties and amounts of ingested foods than food frequency questionnaires^[18]^. The participants
120 were then divided into quintiles (Q1–Q3) based on their dietary vitamin E consumption.
121 Covariates
122 Standardized questionnaires were administered to obtain socio-demographic and lifestyle
123 information. The Mobile Examination Center furnished the results of the examinations, including
124 body mass index (BMI), blood pressure, and various biochemical parameters. Different potential
125 covariates were evaluated using the available literature ^[19–22]^, including age, sex, race/ethnicity,
126 education level, marital status, family income, smoking status, physical activity, BMI, laboratory
127 parameters (total cholesterol and glycated hemoglobin A1c), and comorbidities (e.g., diabetes,
128 hypertension, and cardiovascular disease [CVD]). For detailed information regarding these variables,
129 such as the methods used for measurement, questionnaire data, and the list of variables, please refer
130 to the official NHANES website [(w](http://www.cdc.gov/nchs/nhanes/)w[w.cdc.gov/nchs/nhanes/](http://www.cdc.gov/nchs/nhanes/) accessed on 1 May 2022). The
131 categorization of family income was based on the poverty income ratio (PIR) into three groups: low
132 (PIR ≤ 1.3), medium (PIR > 1.3–3.5), and high (PIR > 3.5). Smoking status was classified according to
133 established definitions from previous studies: never smoked, current smoker, and former smoker. To
134 evaluate physical activity levels, participants were asked about intense exercises leading to notable
135 changes in breathing and heart rate (such as swimming or high-speed cycling), as well as moderate
136 exercises causing mild-to-moderate increases in these physiological parameters (for example, golfing
137 or leisurely biking). The duration of each activity should have been at least 10 min within the last
138 month. Physical activity levels were categorized into three groups: below-moderate (lacking both
139 moderate and intense activities), moderate (absence of intense exercise but inclusion of at least one
140 moderately active pursuit), and high-intensity (presence of at least one episode involving rigorous
141 exertion).
142 The identification of previous illnesses (such as hypertension and diabetes) was established through a
143 questionnaire by asking participants if they had previously informed a doctor about their medical
144 conditions. The history of cardiovascular disease was determined based on individuals' self-reported
145 experiences with congestive heart failure, coronary heart disease, angina pectoris, heart attack, and
146 stroke. BMI was calculated using a conventional method that considers weight and height.

147

148 Statistical analysis
149 This study used a rigorous and comprehensive approach to assess the effects of dietary vitamin E
150 intake on PAD. Analysis of the association between dietary vitamin E intake and PAD was conducted
151 using robust statistical techniques, such as binary logistic regression models that provide odds ratios
152 and 95% confidence intervals, consideration of confounders based on expert judgment, existing
153 scientific literature, and identification of significant covariates in univariate analysis, indicating a
154 thoughtful approach to controlling potential sources of bias. Additionally, the use of a subgroup
155 analysis to examine the relationship between vitamin E intake and PAD within specific subgroups adds
156 to these findings. The handling of missing data through listwise deletion, sensitivity analyses to assess
157 the robustness of the findings, and the reporting and comparison of effect sizes and p-values from
158 various association inference models further enhanced the credibility and reliability of the study's
159 conclusions. Overall, the methodology and analytical approach contributed to the robustness and
160 validity of the findings.
161 Statistical analyses were performed using R Statistical Software (Version 4.2.2, http://www.R-
162 project.org, The R Foundation) and the Free Statistics Analysis Platform (Version 1.9, Beijing, China,
163 [http://www.clinicalscientists.cn/freestatistics).](http://www.clinicalscientists.cn/freestatistics)) A detailed explanation of their functionalities ensured
164 transparency and reproducibility of the analytical methods used in this study. Overall, the statistical
165 analysis appears to be comprehensive, well executed, and supported by appropriate software tools,
166 contributing to the strength of the study's findings.

## 167 Results

168 Characteristics of the study population
169 In total, 31,126 individuals participated in the in-home interviews. Among them, 21,156 participants
170 aged < 40 years were excluded. Furthermore, participants who had missing bilateral ABI data
171 (n=2408), ABI values exceeding 1.4 (n=65), or lacking diet information (n=169) were excluded.
172 Participants with incomplete covariate data were excluded (n=740), resulting in a final sample size of
173 6,588 participants (Fig 1). The study included qualified individuals aged 40 years or older with a PAD
174 prevalence rate of 5.9%. Table 1 presents the basic features of the participants categorized according
175 to their dietary consumption of vitamin E. The three groups differed in terms of sex, age, education
176 level, race, MS, PIR, physical activity, HbA1c, smoking status, hypertension, CVD, and diabetes
177 mellitus ( P < 0.05). However, the distribution of patient characteristics, such as BMI and total
178 cholesterol, among the dietary vitamin E intake groups was similar (P > 0.05)(Table 1).
179 Association between dietary vitamin E intake and PAD
180 In the multivariable logistic regression analyses, it was found that participants in the Q3 group
181 exhibited a lower probability of PAD (OR=0.68, 95% CI=0.51–0.91, P=0.009) than the Q1 group, after
182 adjusting for potential confounders (Table 2, model 5).
183 The adjusted smoothed plots suggested a straightforward linear relationship between dietary vitamin
184 E intake and PAD (Figure 2; P for nonlinearity = 0.897; the highest and lowest 0.5% were trimmed for
185 each dietary vitamin E intake measure). As the dietary vitamin E intake increased, the risk of PAD
186 decreased.
187 Figure 2 shows the correlation between dietary vitamin E intake and PAD in both males and females.
188 Data were analyzed using a multivariate logistic regression model with restricted cubic splines. To
189 account for various factors, such as sex, age, BMI, severity of illness, and reason for admission (Model
190 5), dietary vitamin E intake was considered a continuous variable. The reference standard was the
191 median dietary vitamin E intake. Shaded areas represent 95% confidence intervals. The curves
192 highlight the inverse relationship between dietary vitamin E intake and PAD when considering low or
193 near-median values of dietary vitamin E intake. Owing to limited patient numbers and the nature of
194 the cubic fit analysis, there was significant variability in the 95% confidence intervals at extreme
195 values.
196 Stratification analysis
197 In this study, we performed a subgroup analysis based on various factors, including age, sex, ethnicity,
198 presence of hypertension or diabetes, educational background, history of CVD, income level (PIR),
199 physical activity level, and smoking status. Additionally, sensitivity and supplementary analyses were
200 conducted, and the results of the subgroup analysis indicated no significant evidence of effect
201 modification or interaction based on common risk factors for PAD (all P values for interaction
202 were >0.05) (Fig 3).

203

## 204 Discussion

205 To the extent of our understanding, in a retrospective cross-sectional study conducted on a significant
206 number of participants involved in the NHANES between 1999 and 2004 in the United States, we
207 consistently observed an inverse correlation between the intake of dietary vitamin E and likelihood of
208 developing PAD. Results were consistent across clinical subgroups and in sensitivity analyses. These
209 observations would have important implications for current PAD management strategies.
210 Vitamin E, a membrane-bound antioxidant found in nuts, vegetable oils, and fish, is associated with
211 reduced cellular damage in cardiovascular disease^[23]^. Its antioxidant and anti-inflammatory
212 properties, along with its ability to prevent platelet aggregation and smooth cell proliferation, have
213 been shown to exhibit effects that counteract the development of atherosclerosis ^[24]^.
214 Our research findings align with previous observational studies indicating an inverse relationship
215 between the dietary intake of vitamin E and the occurrence of PAD among participants in the
216 NHANES conducted from 1999 to 2004^[14]^. According to a study conducted by Lane et al.^[23]^, an
217 increased intake of vitamin E exhibited a notable safeguarding effect, regardless of the presence of
218 conventional cardiovascular risk factors, within the population of the United States. Not come singly
219 but in pairs, Antonelli-Incalzi et al.^[20]^ conducted a study on the nutrient consumption of 1251
220 individuals living in their own homes, who were part of the InCHIANTI research. The average age of
221 participants was 68 years. They also investigated whether there was a potential decrease in the risk of
222 PAD associated with a daily intake of Vitamin E equal to or greater than 7.726 mg/d. (OR: 0.37; 95% CI
223 0.16–0.84). In addition, the Rotterdam Study found that there was a negative correlation between
224 vitamin E consumption and PAD in men. However, it remains unclear whether the association
225 between antioxidant intake and PAD differs between sexes or whether these findings are influenced
226 by the different dietary patterns observed in men than in women ^[25].^ Further research is necessary to
227 validate our results and to investigate the intricate relationships and potential mechanisms involved.
228 Recent studies have provided evidence linking dietary vitamin E intake to PAD. Our findings support
229 and expand upon previous research conducted on participants in the NHANES from 1999 to 2004 in
230 the United States. We found that a higher intake of dietary vitamin E was associated with a reduced
231 risk of PAD after accounting for all relevant factors (OR, 0.981; 95% CI, 0.957–1.004). When we
232 divided dietary vitamin E intake into quartiles, individuals in the third quartile had a lower incidence
233 rate of PAD than those in the first quartile (OR, 0.68; 95% CI: 0.51, 0.91). Subgroup analysis yielded
234 similar results without any significant differences (all P values for interactions were >0.05). Further
235 investigations are necessary to validate our findings and explore the intricate relationships and
236 potential mechanisms involved.
237 This was a well-designed retrospective cross-sectional study with a robust sample size and stringent
238 inclusion and exclusion criteria. Baseline data on a wide range of lifestyle factors, including dietary
239 cofactors, were collected, enabling adjustment for potential confounders in the analyses. Additionally,
240 a comprehensive set of sensitivity analyses was conducted to enhance the interpretation of the
241 results and validate the main findings.
242 However, this study had several limitations. First, owing to the cross-sectional and observational
243 nature of the analysis, causal relationships among dietary vitamin E intake, covariates, and PAD could
244 not be definitively established. Despite efforts to control for relevant confounders in the multivariate
245 model, the presence of unmeasured or unknown residual confounders, such as dietary habits and
246 family income, may have led to an overestimation of the observed associations. Nonetheless, the
247 study mainly included American adults, which limits the generalizability of the findings and
248 necessitates further research on diverse populations.

## 249 Conclusions

250 Our study provides supporting evidence indicating a detrimental correlation between the
251 consumption of dietary vitamin E and the occurrence of PAD. Therefore, individuals with insufficient
252 dietary vitamin E intake, especially those with a very low vitamin E intake, should consider increasing
253 their vitamin E intake to lower the risk of developing PAD. These findings should be considered while

272

| 254 | formulating clinical guidelines. |
| --- | --- |
| 255 | **Statements** |
| 256  257 | **Acknowledgement** |
| 258 | We express our gratitude to Jie Liu from the Department of Vascular and Endovascular Surgery at |
| 259 | Chinese PLA General Hospital for providing valuable assistance in statistical analysis, study design |
| 260 | consultations, and offering insightful feedback on the manuscript. |
| 261  262 | **Statement of Ethics** |
| 263 | Study approval statement: The study did not require any additional institutional review board |
| 264 | approval for the secondary analysis, thus ethical review and approval were exempted. |
| 265 | Consent to participate statement: The NHANES study was approved by the Ethics Review |
| 266 | Committee of the National Center for Health Statistics (NCHS), and prior to their involvement, all |
| 267  268 | participants provided written consent after being fully informed. |
| 269  270 | **Conflict of Interest Statement** |
| 271 | The authors have no conflicts of interest to declare. |
| 273 | **Funding Sources** |
| 274 | This study was not financially supported by any funding agencies in the public, commercial, or non- |
| 275 | profit sectors. |
| 276 |  |
| 277 | **Author Contributions** |
| 278 | Conceptualization, Qiang Liu and Jianjun Shi; Data curation, Qiang Liu and Xing Wu; Formal analysis |
| 279 | and Methodology, Qiang Liu, Xing Wu and Jianjun Shi; Funding acquisition, Yun Wang and Jianjun Shi; |
| 280 | Writing—original draft, Qiang Liu and Xing Wu; Writing—Xiang Wang and Fei Zhao; All the authors |
| 281 | have carefully reviewed and given their consent to the final version of the manuscript. |

282

## 283 Data Availability Statement

284 Publicly accessible datasets for this study can be found online. The name of the
285 repository/repositories and their corresponding accession numbers are provided at
286 <http://www.cdc.gov/nchs/nhanes/> (accessed on 1 May 2022).

# References

1. Poledniczek M, Neumayer C, Kopp CW, Schlager O, Gremmel T, Jozkowicz A, et al. Micro- and Macrovascular Effects of Inflammation in Peripheral Artery Disease—Pathophysiology and Translational Therapeutic Approaches. Biomedicines. 2023;11:2284.
2. Shu J, Santulli G. Update on peripheral artery disease: Epidemiology and evidence-based facts. Atherosclerosis. 2018;275:379–81.
3. Hirsch AT, Haskal ZJ, Hertzer NR, Bakal CW, Creager MA, Halperin JL, et al. ACC/AHA 2005 Guidelines for the Management of Patients With Peripheral Arterial Disease (Lower Extremity, Renal, Mesenteric, and Abdominal Aortic): Executive Summary A Collaborative Report From the American Association for Vascular Surgery/Society for Vascular Surgery,⁎⁎AAVS/SVS when Guideline initiated, now merged into SVS Society for Cardiovascular Angiography and Interventions, Society for Vascular Medicine and Biology, Society of Interventional Radiology, and the ACC/AHA Task Force on Practice Guidelines (Writing Committee to Develop Guidelines for the Management of Patients With Peripheral Arterial Disease). Journal of the American College of Cardiology. 2006;47:1239–312.
4. Correction to: 2024 Heart Disease and Stroke Statistics: A Report of US and Global Data From the American Heart Association. Circulation. 2024;149:e1164.
5. Krzyzanowska K, Mittermayer F, Krugluger W, Schnack C, Hofer M, Wolzt M, et al. Asymmetric dimethylarginine is associated with macrovascular disease and total homocysteine in patients with type 2 diabetes. Atherosclerosis. 2006;189:236–40.
6. Tzoulaki I, Murray GD, Lee AJ, Rumley A, Lowe GDO, Fowkes FGR. C-reactive protein, interleukin- 6, and soluble adhesion molecules as predictors of progressive peripheral atherosclerosis in the general population: Edinburgh Artery Study. Circulation. 2005;112:976–83.
7. Joosten MM, Pai JK, Bertoia ML, Rimm EB, Spiegelman D, Mittleman MA, et al. Associations

between conventional cardiovascular risk factors and risk of peripheral artery disease in men. JAMA. 2012;308:1660–7.

11

8. Navab M, Ananthramaiah GM, Reddy ST, Van Lenten BJ, Ansell BJ, Fonarow GC, et al. The oxidation hypothesis of atherogenesis: the role of oxidized phospholipids and HDL. J Lipid Res. 2004;45:993–1007.
9. Bieri JG, Corash L, Hubbard VS. Medical uses of vitamin E. N Engl J Med. 1983;308:1063–71.
10. Colombo ML. An update on vitamin E, tocopherol and tocotrienol-perspectives. Molecules. 2010;15:2103–13.
11. Gahche J, Bailey R, Burt V, Hughes J, Yetley E, Dwyer J, et al. Dietary supplement use among U.S. adults has increased since NHANES III (1988-1994). NCHS Data Brief. 2011;1–8.
12. Fan H, Zhou J, Huang Y, Feng X, Dang P, Li G, et al. A Proinflammatory Diet Is Associated with Higher Risk of Peripheral Artery Disease. Nutrients. 2022;14.
13. Zipf G, Chiappa M, Porter KS, Ostchega Y, Lewis BG, Dostal J. National health and nutrition examination survey: plan and operations, 1999-2010. Vital Health Stat 1. 2013;1–37.
14. Naqvi AZ, Davis RB, Mukamal KJ. Nutrient intake and peripheral artery disease in adults: key considerations in cross-sectional studies. Clin Nutr. 2014;33:443–7.
15. Min J-Y, Cho J-S, Lee K-J, Park J-B, Park S-G, Kim JY, et al. Potential role for organochlorine pesticides in the prevalence of peripheral arterial diseases in obese persons: results from the National Health and Nutrition Examination Survey 1999-2004. Atherosclerosis. 2011;218:200–6.
16. Selvin E, Köttgen A, Coresh J. Kidney function estimated from serum creatinine and cystatin C and peripheral arterial disease in NHANES 1999-2002. Eur Heart J. 2009;30:1918–25.
17. Wu Z, Ruan Z, Liang G, Wang X, Wu J, Wang B. Association between dietary magnesium intake and peripheral arterial disease: Results from NHANES 1999-2004. PLoS One. 2023;18:e0289973.
18. Subar AF, Kipnis V, Troiano RP, Midthune D, Schoeller DA, Bingham S, et al. Using intake biomarkers to evaluate the extent of dietary misreporting in a large sample of adults: the OPEN study. Am J Epidemiol. 2003;158:1–13.

12

19. Amer M, Narotsky DL, Qayyum R. 25-Hydroxyvitamin D and ankle-brachial blood pressure index in adults without peripheral artery disease. Clin Transl Sci. 2014;7:391–5.
20. Antonelli-Incalzi R, Pedone C, McDermott MM, Bandinelli S, Miniati B, Lova RM, et al. Association between nutrient intake and peripheral artery disease: Results from the InCHIANTI study. Atherosclerosis. 2006;186:200–6.
21. Donnan P, Thomson M, Fowkes F, Prescott R, Housley E. Diet as a risk factor for peripheral arterial disease in the general population: The Edinburgh Artery Study. The American Journal of Clinical Nutrition. 1993;57:917–21.
22. Ogilvie RP, Lutsey PL, Heiss G, Folsom AR, Steffen LM. Dietary intake and peripheral arterial disease incidence in middle-aged adults: the Atherosclerosis Risk in Communities (ARIC) Study. Am J Clin Nutr. 2017;105:651–9.
23. Lane JS, Magno CP, Lane KT, Chan T, Hoyt DB, Greenfield S. Nutrition impacts the prevalence of peripheral arterial disease in the United States. Journal of Vascular Surgery. 2008;48:897-904.e1.
24. Kumar M, Deshmukh P, Kumar M, Bhatt A, Sinha AH, Chawla P. Vitamin E Supplementation and Cardiovascular Health: A Comprehensive Review. Cureus [Internet]. 2023 [cited 2024 Mar 10]; Available from: https://[www.cureus.com/articles/184881-vitamin-e-supplementation-and-](http://www.cureus.com/articles/184881-vitamin-e-supplementation-and-) cardiovascular-health-a-comprehensive-review
25. Klipstein-Grobusch K. Dietary Antioxidants and Peripheral Arterial Disease : The Rotterdam Study. American Journal of Epidemiology. 2001;154:145–9.

# Figure Legends

Fig. 1.	Research flowchart

Fig. 2.	Association between dietary vitamin E intake and PAD odds ratio. Solid and dashed lines represent the predicted value and 95% confidence intervals. They were adjusted for age, sex, race/ethnicity, education level, marital status, family income, smoking status, physical activity, BMI, total cholesterol, glycated hemoglobin A1c, and comorbidities. Only 99% of the date is shown.

13

Fig. 3. The relationship between dietary vitamin E intake and PAD according to basic features. Except for the stratification component itself, each stratification factor was adjusted for all other variables.

14

Figure 图	[Click here to access/download;Figure;figure and table.docx](https://www2.cloud.editorialmanager.com/pone/download.aspx?id=36588166&guid=a610e460-01d1-4024-af3c-6ac85314159d&scheme=1)[<https://www2.cloud.editorialmanager.com/pone/download.aspx?id=36588166&guid=a610e460-01d1-4024-af3c-6ac85314159d&scheme=1>](https://www2.cloud.editorialmanager.com/pone/download.aspx?id=36588166&guid=a610e460-01d1-4024-af3c-6ac85314159d&scheme=1)

Fig 1. Research flowchart

Table 1. Baseline characteristics stratified by the dietary vitaminE intake quartiles (Q)**.**

| Variables | Total (n =  6588) | | | | Q1 (n = 2191) | | Q2 (n = 2195) | | Q3 (n = 2202) | p- Value | |
| --- | --- | --- | --- | --- | --- | --- | --- | --- | --- | --- | --- |
| Gender, n | (%) | |  |  |  | |  |  |  | < | 0.001 |
| Male |  | | 3362 | (51.0) | 951 (43.4) | | 1086 | (49.5) | 1325 (60.2) |  |  |
| Female | | | 3226 | (49.0) | 1240 | (56.6) | 1109 | (50.5) | 877 (39.8) |  |  |
| Age (years) | | | 60.1 | ± 13.0 | 61.6 | ± 12.9 | 60.2 | ± 13.2 | 58.4 ± 12.6 | < | 0.001 |
| Race, n (%) | | |  |  |  |  |  |  |  | < | 0.001 |
| Mexican American | | | 1371 (20.8) | | 507 (23.1) | | 449 (20.5) | | 415 (18.8) | | |
| Other Hispanic | | | 261 ( 4.0) | | 93 (4.2) | | 84 (3.8) | | 84 (3.8) | | |
| Non-Hispanic | | white | 3635 | (55.2) | 1070 (48.8) | | 1238 (56.4) | | 1327 (60.3) | | |
| Non-Hispanic | | black | 1127 | (17.1) | 453 (20.7) | | 355 (16.2) | | 319 (14.5) | | |

Other races	194 ( 2.9)	68 (3.1)	69 (3.1)	57 (2.6)

Education level,, n (%)	< 0.001

275 (12.5)

| Less | than high school |  | 1140 | (17.3) | 513 | (23.4) | 352 | (16) |
| --- | --- | --- | --- | --- | --- | --- | --- | --- |
| High | school diploma or | GED | 2562 | (38.9) | 908 | (41.4) | 854 | (38.9) |
| More than high school | | 2886 | | (43.8) 770 (35.1) 989 (45.1) | | | | |
| Married/Living with | | 4381 | | (66.5) 1316 (60.1) 1476 (67.2) | | | | |

800 (36.3)

1127 (51.2)

MS, n (%)	< 0.001

1589 (72.2)

partner

| Widowed/Divorced/Separated 1839 (27.9) 746 | | | | (34) | 604 | (27.5) | 489 | (22.2) |  |  |
| --- | --- | --- | --- | --- | --- | --- | --- | --- | --- | --- |
| Never married 368 ( 5.6) 129 | | | | (5.9) | 115 | (5.2) | 124 | (5.6) |  |  |
|  | | | | | | | | | | |
| Low income | 1504 | (22.8) | 635 | (29) | 490 | (22.3) | 379 | (17.2) | | |
| Medium income | 2290 | (34.8) | 785 | (35.8) | 764 | (34.8) | 741 | (33.7) | | |

PIR, n (%)	< 0.001

High income	2794 (42.4)	771 (35.2)	941 (42.9)	1082 (49.1)

Physical activity, n (%)	< 0.001

Sedentary	3017 (45.8)	1190 (54.3)	989 (45.1)	838 (38.1)

| Moderate | 2071 | (31.4) | 629 | (28.7) | 706 | (32.2) | 736 | (33.4) |
| --- | --- | --- | --- | --- | --- | --- | --- | --- |
| Vigorous | 1500 | (22.8) | 372 | (17) | 500 | (22.8) | 628 | (28.5) |

| BMI, kg/m2 | | | | | |  | 28.5 ± 5.6 | | 28.3 ± 5.5 | 28.4 ± 5.6 | 28.7 ± 5.6 | | 0.135 |
| --- | --- | --- | --- | --- | --- | --- | --- | --- | --- | --- | --- | --- | --- |
| Total cholesterol, | | | | | | mg/dL | 209.6 ± 41.3 | | 210.9 ± 41.9 | 209.0 ± 41.0 | 208.7 ± 41.0 | | 0.150 |
| HbA1c, % | | | | | |  | 5.8 ± 1.1 | | 5.8 ± 1.2 | 5.8 ± 1.0 | 5.7 ± 1.1 | | 0.003 |
| Diabetes, n (%) | | | | | |  |  | |  |  |  | |  |
| No | | | | | |  | 5742 (87.2) | | 1864 (85.1) | 1921 (87.5) | 1957 (88.9) | |  |
| Yes | | | | | |  | 846 (12,8) | | 327 (14.9) | 274 (12,5) | 245 (11.1) | |  |
| Hypertension, | | | | n | (%) |  | |  |  |  |  | | 0.012 |
| No | | | |  |  | 4251 | | (64.5) | 1361 (62.1) | 1432 (65.2) | 1458 (66.2) | |  |
| Yes | | | |  |  | 2337 | | (35.5) | 830 (37.9) | 763 (34.8) | 744 (33.8) | |  |
| CVD, n | (%) | | |  | | | |  |  | 0.025 | | | |
| No |  | | | 5546 | | | | (84.2) | 1809 (82.6) | 1854 (84.5) 1883 (85.5) | | | |
| Yes |  | | | 1042 | | | | (15.8) | 382 (17.4) | 341 (15.5) 319 (14.5) | | | |
| Smoking, | | n | (%) |  | | | |  |  |  |  |  | < 0.001 |
| Never | |  |  | 3043 | | | | (46.2) | 1011 (46.1) | 1038 (47.3) | 994 | (45.1) |  |
| Former | |  |  | 2288 | | | | (34.7) | 707 (32.3) | 753 (34.3) | 828 | (37.6) |  |
| Current | |  |  | 1257 | | | | (19.1) | 473 (21.6) | 404 (18.4) | 380 | (17.3) |  |

PAD, n (%)	< 0.001

No	6199 (94.1)	2023 (92.3)	2054 (93.6)	2122 (96.4)

Yes	389 ( 5.9)	168 (7.7)	141 (6.4)	80 (3.6)

Values are given as mean ± standard deviation or numbers and percentages. BMI, body mass index; CVD, cardiovascular disease; CRP, C-reactive protein; HbA1c, glycosylated hemoglobin; MS, marital status; PIR, poverty income ratio; PAD, peripheral arterial disease.

Table 2. Odds ratios (ORs) and 95% confidence interval (CI) of the dietary vitaminE intake quartiles for PAD.

Variable VitaminE(mg)	P-value Q1 (0-4.4)	Q2 (4.41-7.69)	P-value Q3 (7.7-97.0)	P-value Trend.test	P-value OR (95%CI)	OR (95%CI)	OR (95%CI)	OR (95%CI)

| Model | 1 | 0.944(0.92~0.969) | | <0.001 | 1 | (Ref) | 0.83 | (0.66~1.04) | 0.108 | 0.45 | (0.35~0.6) | <0.001 | 0.69 | (0.61~0.79) | <0.001 |
| --- | --- | --- | --- | --- | --- | --- | --- | --- | --- | --- | --- | --- | --- | --- | --- |
| Model | 2 | 0.973 | (0.949~0.997) | 0.0279 | 1 | (Ref) | 0.97 | (0.76~1.23) | 0.779 | 0.63 | (0.47~0.84) | 0.002 | 0.81 | (0.71~0.93) | 0.003 |
| Model | 3 | 0.975 | (0.951~0.999) | 0.0406 | 1 | (Ref) | 0.97 | (0.76~1.24) | 0.808 | 0.64 | (0.48~0.86) | 0.003 | 0.82 | (0.71~0.94) | 0.005 |
| Model | 4 | 0.976 | (0.952~1) | 0.0517 | 1 | (Ref) | 0.97 | (0.76~1.24) | 0.804 | 0.65 | (0.48~0.86) | 0.003 | 0.82 | (0.71~0.94) | 0.005 |
| Model | 5 | 0.981 | (0.957~1.004) | 0.1075 | 1 | (Ref) | 1 (0.78~1.28) | | 0.992 | 0.68 | (0.51~0.91) | 0.009 | 0.84 | (0.73~0.97) | 0.016 |

Data are presented as odds ratios, 95% CIs (confidence intervals), and p-value. Model 1 adjusted for none.

Model 2 adjusted for age,gender,race, education level,MS, physical activity.

Model 3 adjusted for age,gender,race, education level,MS, physical activity,PIR,BMI,total cholesterol, HbA1c.

Model 4 adjusted for age,gender,race, education level,MS, physical activity,PIR,BMI,total cholesterol,HbA1c, diabetes,hypertension,CVD. Model 5 adjusted for all covariates.

Fig 2.

Fig 3.
